# Green Processes for Chitin and Chitosan Production from Insects: Current State, Challenges, and Opportunities

**DOI:** 10.3390/polym17091185

**Published:** 2025-04-26

**Authors:** Lisa Mersmann, Victor Gomes Lauriano Souza, Ana Luísa Fernando

**Affiliations:** MEtRICs, CubicB, Departamento de Química, NOVA School of Science and Technology (NOVA FCT), Campus de Caparica, Universidade Nova de Lisboa, 2829-516 Caparica, Portugal; l.mersmann@campus.fct.unl.pt (L.M.); v.souza@fct.unl.pt (V.G.L.S.)

**Keywords:** biopolymer, alternative proteins, green chemistry, sustainability, biomass valorization, circular economy

## Abstract

Chitin and chitosan are valuable biopolymers with various applications, ranging from food to pharmaceuticals. Traditionally sourced from crustaceans, the rising demand for chitin/chitosan, paired with the development of the insect sector, has led to the exploration of insect biomass and its byproducts as a potential source. Conventional processes rely on hazardous chemicals, raising environmental concerns. This critical review evaluates emerging “greener” approaches, including biological methods, green solvents, and advanced processing techniques, for chitin/chitosan production from insect-derived materials such as exuviae and cocoons. Two systematic evaluations are included: (1) a cross-comparison of chitin and chitosan yields across insect life stages and byproducts (e.g., up to 35.7% chitin from black soldier fly (BSF) larval exoskeletons can be obtained) and (2) a stepwise sustainability assessment of over 30 extraction workflows reported across 16 studies. While many are labeled as green, only a few, such as bromelain, lactic acid fermentations, or NADES-based processes, demonstrated fully green extraction up to the chitin stage. No study achieved a fully green conversion to chitosan, and green workflows typically required materials with low fat content and minimal pretreatment. These findings will be useful to identify opportunities and underscore the need to refine greener methods, improve yields, reduce impurities, and enable industrial-scale production, while sustainability data need to be generated.

## 1. Introduction

Food production significantly impacts global environmental change [1]. Recent studies show that the global food system is responsible for approximately 35% of total anthropogenic greenhouse gas (GHG) emissions, with animal-based food production alone contributing 57% of that share [2,3]. These emissions stem from various processes, including land use change (deforestation for grazing or feed crops), enteric fermentation in livestock, synthetic fertilizer application, and fossil fuel use in agricultural machinery and transport [3]. Food production contributes not only to climate change but also to biodiversity depletion, freshwater utilization, the disruption of worldwide biogeochemical cycles, and the modification of terrestrial components [1].

Therefore, there is a need to design and shape sustainable food production systems (to be profitable, providing benefits to society and with a positive or neutral impact on the natural environment) [4,5,6]. On the other hand, currently, over 820 million individuals face food scarcity [1]. Even more suffer from poor dietary habits linked to early mortality and increased illness rates [1]. These nutritional deficits need responses that involve high-quality and sustainable food interventions. Stabilizing ecosystems and achieving UN Sustainable Development Goals requires a shift to nutritious diets based on sustainable food sources (“win-win diets”) [1]. Moreover, given the rapid population expansion, anticipated to reach 9.7 billion by 2050, food scarcity will be a significant obstacle [7]. A particular obstacle involves the provision of adequate protein. Around one billion humans worldwide have insufficient protein intake, which has negative consequences for growth and health [8]. Research indicated a reduction in the protein content in crops when the cultivation occurs under elevated atmospheric CO_2_ concentrations (eCO_2_) [9]. This reduction could lead to an extra burden on the protein supply. Staple grains like wheat, rice, and potatoes, which serve as primary dietary protein sources in several nations, appear to be significantly affected by this impact [9]. The exclusive cultivation of a few plant species results in further complications [8,10].

Insects are a potential sustainable alternative food source. They are already part of the standard diet in different parts of the globe, like Africa, Asia, and Latin America, with over 2000 edible species documented worldwide [11]. If adopted globally, insects could provide a viable solution for food scarcity. In Western countries, insects are already used for feed applications [12]. However, to be used in human consumption, it is challenging because of the disgust around insect consumption in this region of the globe [13]. Nevertheless, social attitudes, preferences, and behavior change over time. As a result, foods once considered unappealing or unconventional often become part of modern diets [14,15].

Insects demonstrate notable efficiency in converting biomass into new food resources, including animal-based protein, with some species requiring significantly less feed than conventional livestock [16]. House crickets (*Acheta domesticus*) have slightly better feed conversion ratios (FCRs), ranging from 1.47 to 1.91 kg of feed per kg of live weight gain compared to 1.72 to 2.31 kg for broiler chickens, depending on diet quality [17]. The black soldier fly (*Hermetia illucens*, BSF) demonstrates a wide FCR range, from as low as 1.58 to 8.90, with the most efficient results achieved when reared on nutrient-rich organic waste streams, underscoring their potential in circular food systems [18]. Mealworms (*Tenebrio molitor*) exhibit a broad range of FCRs, from as low as 1.53 [19] (Bordiean et al., 2022) to as high as 6.05 [20], depending heavily on the type and quality of the provided feed. The wide variation in FCR observed across insect species and production systems highlights the sensitivity of efficiency outcomes to species, substrate type, developmental stage, and rearing conditions. Even with variation across studies, optimized diets for insects consistently result in significantly lower FCR values than those reported for beef, with FCRs ranging from approximately 7.53 ± 1.40 to 8.52 ± 1.22 kg of feed per kg of weight gain, depending on a specific diet composition [21].

Nevertheless, insects are among the most land-efficient animal protein sources, surpassed only by plant-based alternative like soybean curd in terms of land use per unit protein [15]. In addition, they exhibit the advantage of utilizing a broad spectrum of feeds, including waste materials and residual products [22]. However, large industrial operations have relied on specifically cultivated feed rather than waste streams to ensure standardized production scales [15]. Insect production involves lower greenhouse gas emissions and requires less water and land use than conventional livestock farming [15,23]. These efficiencies are attributed to their poikilothermic nature, which reduces energy consumption [15,24,25]. Additionally, insects demonstrate productivity advantages, including rapid growth rates, high fecundity, short maturation times, and efficient feed utilization, with consumption fractions reaching up to 100% [14,15,25].

The composition of insects varies significantly depending on life stage and species. Estimates suggest a protein content ranging from 20% to 70%, with essential amino acids comprising 46% to 96% [26]. The fat content varies from approximately 10% to 50%, and the fiber content ranges between 8.5% and 27% [26]. Insects also contain various minerals (mainly calcium) and vitamins (primarily b-complex vitamins) [26]. Furthermore, insects contain bioactive peptides derived from proteolysis that have the potential for medical applications. While in vivo studies are limited, those published highlight various potential features, including blood pressure-regulating, weight-control, antioxidant, inflammation-reducing, antimicrobial, and immune system-regulating attributes [24].

Beyond their nutritional profile, insects present another interesting aspect: they contain chitin. After cellulose, chitin ranks as the second most abundant biopolymer, a β(1→4)-linked glycan [27,28]. The chitin content in insects varies significantly, ranging from approximately 4.5% to 50% of its dry weight, depending on species and development stages [29,30]. Determining chitin content is prone to methodological differences, contributing to further discrepancies in reported values [31]. Physically, chitin is distributed across various structures in the insect, including the exoskeleton, tracheal system, gut lining, and specialized formations like cocoons and spines, reflecting its structural and protective roles [32].

When chitin is deacetylated, chitosan forms as a composite of chitin. Both biopolymers and possible modifications show promising applications in various industries, such as pharmaceuticals, biomedicals, cosmetics, food, packaging, beverages, and agriculture [33]. Chitin and chitosan are built with varying contents of 1→4 glycosidic bonds linking 2-amino-2-deoxy-D-glucopyranose to N-acetyl-2-amino-2-deoxy-D-glucopyranose [34]. Chitin and chitosan differ in physicochemical properties, primarily driven by their polymeric form, degree of deacetylation or acetylation, and molecular weight [34]. It is possible to manipulate and harness attributes such as crystallinity, solubility, and hydrophilicity. However, until now, precise control has been challenging to achieve, and undesired characteristics commonly result from the production process [29,35].

Traditionally, chitin extraction mainly relied on crustacean waste [36]. Due to the rising demand for chitin and chitosan and the general increase in insect production, insects and their chitin-rich byproducts should be considered an alternative chitin source. In addition, the chitin/chitosan sector and the edible insect market have grown. The estimated market value of the global chitin and chitosan market was approximately USD 7.9 billion in 2023, with a forecasted value of USD 24.9 billion by 2030 [34,35]. The combination with a projected compound annual growth rate (CAGR) of 15.3% from 2023 to 2030 highlights its potential [37,38].

By 2030, the European insect sector is projected to produce 1 million metric tons of insect biomass annually [39]. For example, BSF larvae, which contain approximately 30% dry matter and a chitin content of 7–10% of that dry mass [40], a projected biomass output of 1 million metric tons annually in Europe could yield an estimated 21,000 to 30,000 metric tons of chitin. This does not include additional chitin-rich byproducts such as dead adults, pupal exuviae, or sheddings, suggesting even greater resource potential.

Thus, insects and especially their chitin-rich byproducts should be considered an alternative chitin source. While several reviews exist on crustacean-based chitin extraction, few focus on insect-derived chitin, particularly with respect to life stage comparison, sustainability assessment, and waste material utilization. To our knowledge, this is the first review to address these aspects in such a comprehensive and systematic manner.

Therefore, this review aims to (1) systematically compare chitin and chitosan yields across insect life stages and byproducts, (2) evaluate reported green extraction workflows across over 30 processes from 16 studies, and (3) assess sustainability considerations, including solvent recovery, recycling potential, and the presence or absence of standardized metrics. Additionally, methods for chitin quantification are reviewed to identify inconsistencies and their potential impact on reported yields. Since few green methods have been directly applied to insects, promising approaches from other matrices are also discussed. This critical analysis highlights key knowledge gaps and supports the development of insect-based chitin/chitosan production using greener methods, offering valuable guidance for future circular bioeconomy strategies.

## 2. Chitin Composition and Variability Across Insect Species and Residues

**Figure 1:**
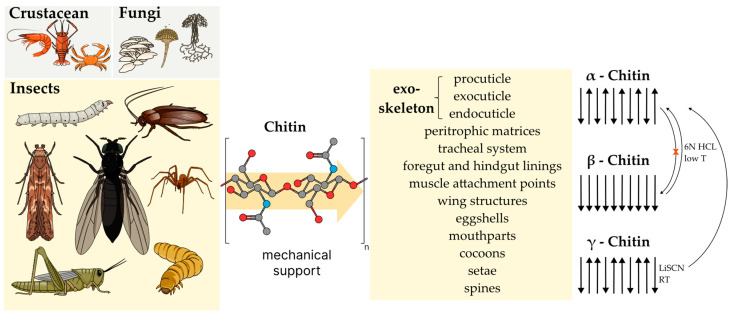
Chitin: Its distribution and conformation in insects (LiSCN—lithium thiocyanate, RT—room temperature, and T—temperature). β- and γ-chitins are transformable into α-chitin but converting α-chitin back into the β-form has not been achieved.

Chitin primarily exists in the exoskeletons of arthropods, fungal cell walls, and nematodes [41]. In the insect body, chitin is found in several layers of the exoskeleton, such as in the newly formed procuticle and the exo- and endocuticle [41,42]. Its principal function is mechanical protection and support for the insect by creating a light, strong, and structured material for their exoskeletons (Figure 1). Cuticle proteins linked to chitin are primarily responsible for the mechanical attributes of the cuticle [41].

Chitin is used in the peritrophic matrices of insects, where it helps create a permeable membrane connecting the midgut epithelium with the food bolus to improve digestion [41]. It also protects against mechanical disturbance and, interestingly, the impact of toxins and pathogens [41]. However, it is absent from the external barrier layer, the epicuticle [41,42]. Chitin can be found in insects within the tracheal system, foregut and hindgut linings, in muscle attachment points, wing structures, eggshells and mandibles, other mouthparts, and specialized structures such as cocoons and setae and spines [32,43,44,45,46,47,48].

Other components, including minerals such as calcium carbonate or pigments, and catechol, are incorporated into the protein–chitin structure [49]. Studies indicate that chitin polymers are organized in microfibrils of approximately 3 nm in width [41]. These microfibrils are stabilized by hydrogen bonds between amine and carbonyl groups [41].

While these microstructural elements define the internal architecture of chitin, the material’s outward surface morphology is equally important and exhibits notable variability. The surface morphology of insect-derived chitin can vary significantly, not only between species, but also across different anatomical regions and life stages [50,51]. Scanning electron microscopy (SEM) analyses have demonstrated this anatomical diversity in chitin organization, revealing distinctive nanoscale architectures in cuticular regions such as wings, eyes, legs, and thorax, with implications for their mechanical and functional properties [50].

Adding another layer of complexity, insect chitin also exists in different polymeric forms based on the arrangement of linear polysaccharides: α-chitin forms when chains converge, with neighboring piles aligned in opposite directions (antiparallel), meaning that chains are “up” and “down” [47]. Conversely, β-chitin consists of neighboring piles with chains aligned in the same direction, such as all “up”. A combination of both is described as γ-chitin. It is suggested that, in γ-chitin, every third layer has an opposite direction (Figure 1) [52]. The α-chitin form predominates [41] and is [40] characterized by its high degree of crystallinity due to the antiparallel orientation of the chains [41,53]. A comparatively packed arrangement with stronger hydrogen bonding results [54]. In contrast, both β- and γ-chitin are often involved in forming more pliable morphologies [41].

For example, β-chitin was identified in the cocoons of figwort weevil [47], while γ-chitin was determined in the cocoons of the Australian spider beetle (*Ptinus tectus*) [40]. The latter was also identified in the larvae of the sawfly (*Phymatocera aterrima*), the silkworm (*Antheraea perny*) [40], and the cocoon of the Dusky Tussock moth (*Orgyia dubia*) [55].

β- and γ-chitins are transformable into α-chitin. At room temperature, γ-chitin easily changes to the α-form in a saturated lithium thiocyanate medium [47]. β-chitin, with lower crystallinity, easily shifts to the α-formation at low temperatures in 6N hydrochloric acid [47]. β-chitin with higher crystallinity undergoes a more challenging transformation to the α form [47]. It has been suggested that converting α-chitin back into the β-form is impossible [56]. β-chitin is an interesting allomorph characterized by enhanced solubility and greater affinity towards solvents and swelling [57,58]. It exhibits significantly diminished intermolecular hydrogen bonds stemming from the uniform alignment of its polymer strands [58], in contrast to α-chitin. These polymorphic transitions may also be influenced by underlying thermodynamic factors. For example, inverse temperature dependence observed in chitin systems has been attributed to hydrophobic interactions, resembling low critical solution temperature phase behavior [59]. However, the detailed mechanisms governing these structural conversions remain an area of ongoing research.

The effective use of chitin requires understanding the complex insect matrix, which changes during different life stages. In general, insect materials with structural or protective functions, such as exuviae, pupal shells, and cuticles, tend to exhibit higher chitin content, while whole-body samples from larvae or adults display more variability due to differences in biomass composition, developmental stage, and species (see Table 1). Depending on the stage, variations can occur in chitin content, the degree of deacetylation, molecular weights, viscosity, crystallinity, and surface morphology [60]. In addition to these factors, mineral content also plays a crucial role towards the structure and properties of chitin during different developmental stages or species. It plays a significant role in the biomineralization process and the formation of fibrous and porous structures of chitin [61]. Nevertheless, although chitin is a key component in biomineralized structures in mollusks and crustaceans, such mineralization is rare in insects, and its structural and biochemical role in this context remains an ongoing area of research [62]. Mineral content in insects varies significantly across developmental stages and species. In the case of BSF, the mineral content exhibits variability across developmental stages, ranging from 8.83% to 9.56% [63]. Magnesium and calcium salts, with carbonate as the main counter-anion, were the primary minerals detected in the ash analysis [64]. In contrast, mealworms have significantly lower mineral content, approximately 2 to 3% [65]. However, insects generally contain less minerals than crustaceans (30% to 50%) [66].

Another distinction between insects and crustaceans is the high content of melanin in insects, which is bound to chitin [34]. A colorless product can be obtained with an extensive bleaching step. In crustaceans, carotenoids are present instead of melanin. These carotenoids can be recovered during the chitin extraction process as a secondary product [57]. The additional process leads to an economically attractive crustacean extraction procedure, with astaxanthin as one of the main extracted carotenoids. Future research should explore the potential value of melanin pigments in insects and evaluate their utility as a valuable resource to enhance the economic feasibility of chitin extraction from insects.

Returning to the biopolymer of interest in this review, chitin faces significant challenges due to its insolubility in standard inorganic and organic solvents (except for concentrated mineral acids) [67,68]. This insolubility arises from strong hydrogen bonding and extensive crystallinity [67,68]. Hence, chitin is frequently converted into chitosan via deacetylation. Chitosan is soluble in moderately acidic solutions [69]. The improved solubility of chitosan is due to a higher abundance of free primary amine groups [69]. To some extent, it is also influenced by a reduced molecular weight [69]. Chitosan’s superior solubility and intrinsic antimicrobial activity make it useful in various fields. It is a valuable resource due to its non-toxicity, antibacterial attributes, film-forming properties, and excellent biodegradability [67,70,71]. Combining these characteristics leads to promising food, packaging, water treatment, pharmaceuticals, and more applications. It is applied in innovative food packaging materials, where its properties help extend shelf life and reduce food spoilage [72,73]. Chitosan is also used in wound-healing materials [74].

The differentiation between chitin and chitosan relies on the degree of acetylation within the biopolymer. It is categorized as chitin when the N-acetyl group content exceeds 50%, while it is considered chitosan when it falls below 50% [69]. The degree of deacetylation is commonly used to characterize the biopolymer, describing the percentage of acetyl groups removed from the chitin molecule to form chitosan (see Table 1).

The molecular weight of chitosan obtained from insects ranges from 26 to 300 kDa, but lower molecular weights from 3 to 7 kDa have also been reported [69]. Insect-derived chitosan has a relatively low molecular weight [69]. Primarily, this is due to a lower chitin polymerization degree and more significant overall structural differences [69].

Table 1 summarizes recent studies on extracting and characterizing chitin and its modification to chitosan from insects. The focus is on byproducts. It also highlights studies that analyze the distinct life stages of insects.

For instance, the collected biomass of BSF demonstrates varying chitin content, ranging from 8% to 24% [40]. Sheddings and cocoons are the most abundant sources of chitin in BSF, as indicated in Table 1. These byproducts can be used to obtain chitin. They exhibit the highest dry matter content, with sheddings at around 92% and cocoons at 94% [40]. The high dry matter content can be beneficial, particularly in reducing energy use during drying. However, sheddings are more challenging to purify [40]. Triunfo et al. (2022) further explored BSF by analyzing biomasses from larvae, pupal exuviae, and dead adults [60]. Among these, pupal exuviae yielded the highest chitin content (25.5%) and high purity (86.8% after bleaching), comparable to commercial crustacean-derived chitin. These results indicated the potential of BSF byproducts. The selection of the residues is crucial for the overall chitin yield and its quality.

Given the critical role of accurate chitin/chitosan quantification in assessing process efficiencies and optimization, several analytical techniques have been developed to address these challenges. The quantification of chitin and chitosan yield often involves gravimetric procedures. However, various methods can be applied, frequently resulting in varying results (Table 1). The commonly used gravimetric method may be inaccurate due to remaining impurities in the extracted chitin [49]. D’Hondt et al. (2020) implemented a simplified method for determining the “content and average degree of acetylation of chitin in crude black soldier fly larvae samples” [75]. Another study by Nurfikari and de Boer (2021) compared D’Hondt’s method with electrochemical detection (ECD) to circumvent the need for expensive tandem mass spectrometry (MS/MS) instrumentation [49]. The results showed that the chitin samples may not be as pure as desired. A chitin content of 68% was found in the extracted insect chitin sample [49]. Notably, 89% was recorded for a commercially available shrimp shell chitin sample [49].

Various advanced methods have been employed to enhance chitin determination. One commonly used approach adapts the acid detergent fiber (ADF) method, initially designed for cellulose, by introducing a technique correcting ADF with an additional acid detergent lignin determination (ADF-ADL) to account for the catechol and/or quinone content [76]. Luparelli et al. (2023) developed a quantitative Ultra-Performance Liquid Chromatography method coupled with Electrospray Ionization Mass Spectrometry (UPLC-ESI/MS) specifically for the “simultaneous determination of chitin and protein content in insects” [77]. A new technique utilizing ultra-high-performance liquid chromatography (UHPLC) coupled with fluorescence detection was developed to analyze insect-derived chitin. This innovative approach enhances sensitivity and precision in chitin quantification, making it a valuable tool for addressing challenges associated with impurities and varying results [29]. Recently, Kvasnička et al. (2023) employed electrophoretic determination [78]. An alternative technique utilizing calcofluor staining was suggested by Machałowski et al. (2019) [79] and Henriques et al. (2020) [31].

Together, these advancements in analytical methods underscore the growing emphasis on accurate and efficient quantification techniques to support green chitin extraction processes. In the following chapter, the conventional chemical extraction and deacetylation, as mentioned in Table 1, will be discussed in detail.

**Table 1:** Chitin and chitosan production from different life stages and (mainly) byproducts of insects.

Species	Part	Dry Matter ^a^ [%]	Chitin Yield ^a^ [%]	Chitosan Yield ^a^ [%]	DD Chitosan ^a^ [%]	Info	Ref.
*Hermetia* *Illucens* (Black Soldier Fly, BSF)	Larvae	29.5 ± 0.3	(1.) 9.5 ± 0.6 (2.) 7.8 ± 0.3	n.s.	89 **	**Process:** Conventional chemical extraction and deacetylation. **Methods for chitin/chitosan determination:** (1.) Gravimetric determination of chitin based on dry matter. (2.) Chitin determination based on a calculation including glucosamine concentration via UPLC-MS/MS and acetate concentration via HPLC-RID. Method proposed by D’Hondt et al. (2020) [75]. **Further info:** Cocoon chitin was more crystalline (94%) compared to sheddings (89%). * Second attempt with extensive deproteinization process led to 26.6%. ** After prolonged deacetylation time of 3 h (for a time of 1 h, sheddings showed significantly lower deacetylation reactivity with degree of deacetylation (DD) of 50%).	[40]
	Prepupae	51.5 ± 1.1	(1.) 9.1 ± 0.02 (2.) 10.9 ± 0.7				
	Pupae	60.9 ± 0.9	(1.) 10.3 ± 0.7 (2.) 10.7 ± 0.1				
	Shedding	92.3 ± 0.5	(1.) 31.1 ± 0.3 * (2.) 23.7 ± 1.9				
	Cocoon	94.2 ± 0.9	(1.) 23.8 ± 1.5 (2.) 22.4 ± 0.9				
	Adults	60.5 ± 3.1	(1.) 5.6 ± 0.4 (2.) 8.4 ± 1.9				
BSF	Larvae	n.s.	3.06	n.s.	n.s.	**Process:** Conventional chemical extraction. No chitosan preparation. **Methods for chitin determination:** Gravimetric calculation of chitin based on dry weight. **Further info:** Chitin crystallinity index (CrI) varied in different life stages with 33.09%, 35.14%, 68.44%, and 87.92% for larvae, prepupa, puparium, and adult. Different surface morphologies of chitin observed between life stages.	[80]
	Prepupae	n.s.	3.1	n.s.	n.s.		
	Puparium	n.s.	14.1	n.s.	n.s.		
	Adults	n.s.	2.9	n.s.	n.s.		
BSF	Pupae Exuviae (BSFE)	n.s.	9	n.s.	n.s.	**Process:** Conventional chemical extraction. No chitosan preparation. **Methods for chitin determination:** Gravimetric determination of chitin based on dry weight. **Further info:** Degree of acetylation (DA) affirmed that Pupae Exuviae (115%) ^b^ chitin demonstrated higher purity than Dead Imago (86%) chitin. CrI of chitin varied between Imago and Pupae Exuviae with 25.5% and 49.4%, respectively. BSFE nonporous. BSFI chitin indicates mesoporosity.	[81]
	Dead Imago (BSFI)	n.s.	23	n.s.	n.s.		
BSF	Larval Exoskeletons	n.s.	(1.) 35.7 ± 0.6 (2.) 31	13–47	34–72	**Process:** Larval exoskeletons from protein production. Optimized chemical extraction. Heterogenous and homogeneous chemical deacetylation. Two setups: (1.) small scale and (2.) 10 L-scale. **Methods for chitin/chitosan determination:** Chitin content measured as acid detergent fiber (ADF) subtracting the acid detergent lignin (ADL) according to Hahn et al. (2018) [76]. Chitosan yield based on dried chitin mass. **Further info:** At lower temperatures, deacetylation led to chitosan with lower DD and a solution with higher viscosity. But, in comparison to the chitosan obtained at higher temperatures, the chitosan yield was much lower.	[63]
BSF	Pupal Shell	n.s.	12.4	n.s.	81.5	**Process:** Microbial fermentation for chitin extraction. Conventional chemical deacetylation. **Methods for chitin/chitosan determination:** No info on chitin/chitosan determination. **Further info:** CrI extracted from BSF pupal shell (52.8%) and chitosan (55.4%) corresponded to lower crystalline index values. Chitin and chitosan samples displayed even surface structure with nonporous morphology and ordered hexagonal microfibrils.	[82]
BSF	Pupal Exuviae	n.s.	(1.) 7.78 ± 0.68 to 11.85 ± 1.16 (2.) 10.18 ± 0.42	(1.) n.s. (2.) 6.58	n.s.	**Process:** Two methods: (1.) Biological chitin extraction with different bacteria species. (2.) Conventional chemical extraction. Conventional chemical deacetylation. **Methods for chitin/chitosan determination:** Gravimetric calculation of chitin based on dry weight. Chitosan yields based on dried chitin mass. **Further info:** Low chitosan yield may have been the result of sample loss during deacetylation. Biologically extracted chitin exhibited irregular surfaces with abundant porous fibers. Chitin obtained via chemical method had a smooth surface with repeating circular and hexagonal elements.	[83]
BSF	Instar 3	n.s.	(1.) 7.23 ± 0.33 (2.) 10.2 ± 0.83	n.s.	n.s.	**Process:** Insects fed with two different organic wastes: (1.) Fruit waste. (2.) Vegetable waste. Conventional chemical extraction. No chitosan preparation. **Methods for chitin determination:** Gravimetric determination based on dry weight. **Further info:** CrI varied in life stages (Instar 3, Instar 4, Instar 5, Prepupa, Pupa) and with feed when fruit waste fed as follows: 51.16%, 58.49%, 75.03%, 71.08%, 59.62%, and, when vegetable waste was fed as follows: 62.09%, 57.39%, 51.48%, 75.89%, 71.39%. Samples displayed an uneven and substantial surface structure consisting of pentagonal and hexagonal units, along with microfiber for all extracted samples with a significant surface diversity.	[84]
	Instar 4	n.s.	(1.) 11.01 ± 0.46 (2.) 9.49 ± 0.11	n.s.	n.s.		
	Instar 5	n.s.	(1.) 9.17 ± 0.84 (2.) 9.83 ± 0.19	n.s.	n.s.		
	Prepupa	n.s.	(1.) 11.78 ± 0.13 (2.) 11.78 ± 0.13	n.s.	n.s.		
	Pupa	n.s.	(1.) 6.82 ± 0.36 (2.) 8.66 ± 0.29	n.s.	n.s.		
BSF	Puparia	n.s.	(1.) 25.39 ± 2.43 (2.) 21.19 ± 5.71	n.s.	n.s.	**Process:** Two methods: (1.) Conventional chemical extraction. (2.) ADF and ADL method with H_2_SO_4_ and CTAB, according to Hahn et al. (2018) [76]. No chitosan preparation. **Methods for chitin determination**: (1.) Gravimetric determination based on dry weight. (2.) Acid detergent fiber (ADF) subtracting the acid detergent lignin (ADL) (ADF-ADL) according to Hahn et al. (2018) [76]. **Further info:** Chitin crystallinity index (CrI) varied in various developmental stages and depending on method: Puparia, Flakes Adult insect for chemical method: 74.1%, 61.1%, and 77.8%; and for ADF-ADL method: 70.8%, 50.0%, and 39.0%. All below, commercial shrimp chitin samples. Flake samples exhibited a recurring pattern like honeycombs; pupae shell chitin showed compact surface structures repeating with circular and hexagonal elements; however, adult insect chitin demonstrated lightly arranged oval elements interspersed with circular structures, featuring repeated fiber arrangements and lacking pores. In the case of chitins isolated using the ADF-ADL method, the Puparia and Flake samples exhibited a greater degree of homogeneity, a light not dense powder, in contrast to the samples acquired through the acid-based method.	[85]
	Flakes (from oil production)	n.s.	(1.) 20.69 ± 2.47 (2.) 26.78 ± 2.17	n.s.	n.s.		
	Adult	n.s.	(1.) 7.75 ± 0.49 (2.) 7.94 ± 1.92	n.s.	n.s.		
BSF	Larval Exuviae	n.s.	(1.) 10.9 ± 0.1 (2.) 11.1 ± 0.4	n.s.	n.s.	**Process:** Conventional chemical extraction plus acid hydrolysis (for chitin determination). **Methods for chitin/chitosan determination:** Purity determination with the following method: Quantification of monomers after acidic hydrolysis (glucosamine, N-acetylglucosamine, acetic acid). Two methods were used to quantify glucosamine and N-acetylglucosamine: (1.) LC-ECD and (2.) LC-MS/MS based on D’Hondt et al. (2020) [75]. To quantify acetic acid, LC-UV was employed Chitin determination in a loaded sample via glucosamine, acetate, and acetylglucosamine contents. Gravimetric determination of chitin in insect material based on dry weight corrected with obtained chitin content in analyzed material. **Further info:** Purity: Chitin content measured in extracted chitin samples: larval exuviae: 68.1 ± 0.7%, puparium: 81.7 ± 1.1%, adult flies 79.4 ± 0.5%; commercial shrimp shell chitin: 89.1 ± 0.1%	[49]
	Puparium	n.s.	(1.) 18.5 ± 0.4 (2.) 18.5 ± 0.3	n.s.	n.s.		
	Adult Flies	n.s.	(1.) 9.6 ± 0.2 (2.) 9.2 ± 0.3	n.s.	n.s.		
BSF	Exuviae	8	20	n.s.	n.s.	**Process:** Deproteinization via superheated water hydrolysis. **Methods for chitin determination** Gravimetric determination based on dry weight. **Further info:** Besides chitin, proteins were acquired in an aqueous solution (as opposed to strong alkaline solution) with further usability. Chitin exhibited a nonporous surface morphology composed of orderly repeated hexagons.	[86]
BSF	Larvae	22.0 ± 0.8	(1.) 13 ± 0.7 (2.) 10 ± 0.7	(1.) 25 ± 2.5 (2.) 33 ± 0.4	(1.) 91 (2.) 92	**Process:** Chemical extraction (formic acid, NaOH). Conventional chemical deacetylation. **Methods for chitin/chitosan determination:** Gravimetric determination for yield, ADF-ADL method for content in raw samples. Chitosan yield based on dried chitin mass. **Further info:** Chitin content of raw samples: larvae: 12.4 ± 1.7%, pupal exuviae: 25.5 ± 0.5%, and dead adults: 12.8 ± 1%. Bleached and unbleached products were analyzed: (1.) unbleached and (2.) bleached. CrI for unbleached chitin: larvae: 90.0%, pupal exuviae: 67.0%, and adults: 96.0%; and for bleached chitin: larvae: 84.0%, pupal exuviae: 62.0%, adults: 93.0%; and commercial chitin: 98.0%. Adult chitin showed highest surface complexity (including nanometric and micrometric features), decreasing in pupal exuviae and larvae. Bleaching had little effect on larvae and pupal exuviae but removed round particles in adult chitin. Deacetylation reduced fibrillation of chitin, resulting in a rough but more homogeneous chitosan structure compared to chitin.	[60]
	Pupal Exuviae	94.0 ± 0.7	(1.) 31 ± 1.6 (2.) 23 ± 1.9	(1.) 28 ± 4.5 (2.) 42 ± 1.5	(1.) 83 (2.) 90		
	Dead Adults	93.0 ± 0.9	(1.) 9 ± 0.4 (2.) 6 ± 0.1	(1.) 27 ± 2.0 (2.) 41 ± 1.0	(1.) 91 (2.) 93		
*Zophobas* *morio * (superworm)	Cuticle of Larva	n.s.	11.21 ± 0.55	81.36 ± 1.35	83.57 ± 0.28	**Process:** Conventional chemical extraction and deacetylation. **Methods for chitin/chitosan determination:** Gravimetric chitin determination based on dry weight. Chitosan yield based on dried chitin mass. **Further info:** Relative crystallinity index (RCI) for cuticle of larva chitin: 68.0% and chitosan: 66.3%; for cuticle of adult chitin: 89.2% and chitosan: 80.2%. Both chitin samples exhibited surface morphology with greater density featuring occasional pores and a fibrous structure. Chitosan of the cuticle of the adult demonstrated pores. Chitosan of the cuticle of the larva exhibited a dense morphology, devoid of nanofibers, characterized by pores and repetitive hexagon elements.	[87]
	Cuticle of Adult	n.s.	20.89 ± 0.14	83.42 ± 0.86	88.72 ± 1.13		
*Blaptica* *Dubia* (Dubia Roach)	Cuticle of Nymph	n.s.	19.23 ± 0.60	75.07 ± 0.25	75.75 ± 0.19	**Process:** Conventional chemical extraction and deacetylation. **Methods for chitin/chitosan determination:** Gravimetric chitin determination based on dry weight. Chitosan yield based on dried chitin mass. **Further info:** RCI for cuticle of nymph chitin: 80.9% and chitosan: 66.6%; for cuticle of adult chitin: 86.8% and chitosan: 73.9%. Chitin of the adult’s cuticle demonstrated even surface configuration lacking both pores and nanofibers. Chitin of the cuticle of the nymph showed rough surface morphology with fragmented fibers and no pores. Chitosan samples exhibited an uneven morphology, but, in contrast to chitin, they had fewer fibers.	[87]
	Cuticle of Adult	n.s.	15.68 ± 0.20	75.75 ± 0.45	86.33 ± 3.13		
*Tenebrio molitor* (Meal-worm)	Cuticle of Larva	n.s.	13.25 ± 0.63	74.93 ± 0.93	76.32 ± 0.26	**Process:** Conventional chemical extraction. Conventional chemical deacetylation. **Methods for chitin/chitosan determination:** Gravimetric chitin determination based on dry weight. Chitosan yield based on dried chitin mass. **Further info:** RCI for cuticle of larva chitin: 71.7% and chitosan: 65.3%; for cuticle of adult chitin: 73.3% and chitosan: 67.6%. Both chitin samples demonstrated uneven surface structures with no pores and disrupted fibers. Chitosan also showed an uneven surface; however, it displayed fewer fibers in contrast to chitin. Chitosan from the adult’s cuticle showed pores.	[87]
	Cuticle of Adult	n.s.	15.13 ± 0.78	78.96 ± 0.45	89.21 ± 0.96		
Meal-worm	Cuticles	94.6 ± 0,1	70.9	31.9	53.9	**Process:** Enzymatic deproteinization. Skipped demineralization step due to low mineral concentration (ash content 3.7%). Conventional chemical deacetylation. **Methods for chitin/chitosan determination:** Gravimetric chitin and chitosan determination based on dry weight. Chitosan yields based on dried chitin mass. **Further info:** DD of chitosan was not low enough. CrI of chitin: 53.7%, CrI of chitosan: 30.1%. Chitosan that originated from mealworm’s cuticles exhibited larger elements of varied shapes. Chitin and chitosan showed rougher morphology and nanofiber structures.	[30]
Meal-worm	Larva Protein Extraction Waste	n.s.	n.s.	(1.) ca. 17 * (2.) ca. 22 *	(1.) 82 ± 9.09 (2.) 84 ± 2.94	**Process:** Conventional chemical extraction and deacetylation. Skipped demineralization step (inorganic material only 2–3%). Two batches (1.) and (2.). **Methods for chitin/chitosan determination:** Gravimetric chitin and chitosan determination based on dry weight. Chitosan yield based on dried chitin mass. **Further info:** Samples from molting stage and adults exhibited increased reflection peak intensities in contrast to larval samples, indicating elevated crystallinity levels. Low molecular weights analyzed for all chitosan samples (600–800 kDa for larva and adult samples, even lower results with high variability for molt samples). * Data estimated from figure [58].	[65]
	Waste from Molt	n.s.	n.s.	(1.) ca. 1 * (2.) ca. 4 *	(1.) 83 ± 2.82 (2.) 84 ± 2.16		
	Adult Insects	n.s.	n.s.	(1.) ca. 17 * (2.) ca. 21 *	(1.) 84 ± 4.54 (2.) 81 ± 0.81		
Meal-worm	Larvae	(1.) 97.7 ± 0.05 (2.) 97.7 ± 0.05	(1.) 5.3 ± 0.38 (2.) 6.0 ± 0.10	(1.) 73.9 ± 2.03 (2.) 80.0 ± 0.58	(1.) 67.4 (2.) 70.9	**Process**: Conventional chemical extraction and deacetylation. Two different processing methods: (1.) First, deproteinization, then, demineralization (DEP-DEM). (2.) First, demineralization, then, deproteinization plus decoloring (DEM-DEP). **Methods for chitin/chitosan determination:** Gravimetric determination of chitin based on dry weight. Chitosan yield based on dried chitin mass. **Further info:** CrI for larvae chitin: (1.) 48% and (2.) 52%; chitin adult: (1.) 50% and (2.) 56%. DEM-DEP exhibited lower mineral concentrations and increased viscosity in contrast to the first method and was classed more efficient. In contrast to chitin obtained by DEP-DEM treatment (fibrous and uneven surface), chitin acquired through the DEM-DEP process displayed a more even surface morphology and more evident pores. Larger and more abundant pores were analyzed by chitin of the DEM-DEP method.	[88]
	Adult	(1.) 97.8 ± 0.08 (2.) 97.8 ± 0.15	(1.) 10.9 ± 0.18 (2.) 14.6 ± 0.15	(1.) 81.9 ± 1.36 (2.) 87.3 ± 2.21	(1.) 69.3 (2.) 73.2		
Meal-worm	Larval Exuviae	n.s.	(1.) 7.9 ± 0.1 (2.) 8.6 ± 0.1	n.s.	n.s.	**Process:** Conventional chemical extraction plus acid hydrolysis (for chitin determination). **Methods for chitin/chitosan determination:** Purity determination with the following method: Quantifying monomers after acidic hydrolysis (glucosamine, N-acetylglucosamine, acetic acid). Two methods were used to quantify glucosamine and N-acetylglucosamine: (1.) LC-ECD and (2.) LC-MS/MS based on the study of [75]. To quantify acetic acid, LC-UV was employed. Chitin determination in loaded sample via glucosamine, acetate, and acetylglucosamine contents. Gravimetric determination of chitin in insect material based on dry weight corrected with obtained chitin content in analyzed material. **Further info:** Purity: Chitin content of extracted sample: 54.1 ± 1.2%; commercial shrimp shell chitin: 89.1 ± 0.1%.	[49]
*Bombyx mori* (Silkworm)	Pupae	n.s.	18	91	66.9 ± 0.2	**Process:** Conventional chemical extraction and deacetylation. **Methods for chitin/chitosan determination:** Gravimetric procedure. No detailed info. Chitosan yields based on chitin mass. **Further info:** CrI for pupae chitin: 74.5% and for chitosan: 48.4%; for eggshell chitin: 75.2% and for chitosan: 38.1%. In both chitosan samples, consistent “sheet-like” surface characteristics were observed without fibrous morphology. An increased quantity of “particulate matter” was detected in chitosan from pupae.	[45]
	Egg Shells	n.s.	6	80	59.2 ± 0.2		
Silkworm	Cuticle	n.s.	(1.) 51.93 ± 0.73 (2.) 56.94 ± 4.05	n.s.	n.s.	**Process:** Aim of the work was not the extraction of chitin, but the chitin content of cuticle was determined after chemical deproteinization. Determination based on control sample after (1.) 12 days and (2.) 14 days after the start of the study to determine the effect of jellyfish venom on silkworm cuticle. **Methods for chitin/chitosan determination:** Gravimetric procedure. No detailed info. **Further info:** Not applicable.	[89]
*Acheta domesticus* (House Cricket)	Exuviae from Various Instar Stages	n.s.	(1.) 9.6 ± 0.2 (2.) 9.9 ± 0.2	n.s.	n.s.	**Process:** Conventional chemical extraction plus acid hydrolysis (for chitin determination) **Methods for chitin/chitosan determination:** Purity determination with the following method: quantification of monomers after acidic hydrolysis (glucosamine, N-acetylglucosamine, and acetic acid). Two methods were used to quantify glucosamine and N-acetylglucosamine: (1.) LC-ECD and (2.) LC-MS/MS based on D’Hondt et al. (2020) [75]. To quantify acetic acid, LC-UV was employed. Chitin determination in loaded sample via glucosamine, acetate, and acetylglucosamine contents. Gravimetric determination of chitin in insect material based on dry weight corrected with obtained chitin content in analyzed material. **Further info:** Purity: Chitin content of extracted chitin sample: 66.7 ± 0.3%; commercial shrimp shell chitin: 89.1 ± 0.1%.	[42]

^a^ Numbers (e.g., 1., 2.) refer to methods and processes, and * and ** refer to further info; when used, please refer to the ‘Info’ cell in the corresponding row for specific definitions. ^b^ Authors suggest that the presence of impurities such as water or mineral residues in extracted chitin result in a DA > 100%. n.s.—not stated; CrI—crystallinity index; RCI—relative crystallinity index; DD—degree of deacetylation; DA—degree of acetylation; BSF—black soldier fly; BSFE—BSF—Pupae Exuviae; BSFI—BSF—Dead Imago; LC-ECD—liquid chromatography with electrochemical detection; LC-MS/MS—liquid chromatography-tandem mass spectrometry; LC-UV—liquid chromatography with ultraviolet detection; UPLC-MS/MS—ultra-performance liquid chromatography coupled with tandem mass spectrometry; HPLC-RID—high-performance liquid chromatography with refractive index detection; DEP—deproteinization; DEM—demineralization; ADF—acid detergent fiber; ADL—acid detergent lignin; ADF-ADL—acid detergent fiber subtracting the acid detergent lignin; and CTAB—cetrimonium bromide.

## 3. Common Chitin Extraction and Chitosan Modification Across Different Biomass Sources

The prevalent procedure for extracting chitin from insects typically involves a sequential demineralization, deproteinization, and deacetylation process. This method was initially adapted for crustaceans as the primary raw material. Consequently, most methods focus on crustaceans. However, there is a growing shift towards alternative resources such as insects. Fungal systems are also explored as a potential biomass for chitin and chitosan extraction [90].

To better illustrate the distinctive characteristics and extraction challenges across biomass sources, Table 2 provides a side-by-side comparison of conventional processing workflows for crustaceans, fungi, and insects. The parameters considered include raw material availability, chitin yield, process scalability, and pretreatment requirements.

While this review focuses primarily on insect-based chitin and chitosan production, understanding the broader context of other biomass sources is essential for meaningful comparison. The chitin content in insects has already been discussed in the introduction, with values ranging widely depending on species and life stage (1.2–60%) [91] (see Table 2).

Crustaceans, the traditional industrial source of chitin, typically contain 6–75% chitin in their dry biomass [91,92]. This group benefits from long-established, industrially optimized extraction workflows, which are well documented in the literature [92,93]. Their consistent composition, high chitin content, and predictable supply chains have made them the benchmark for chitin and chitosan production.

In contrast, the chitin content varies significantly among fungal species, from as low as 2% in yeasts to 42% in *Euascomycetes* [94]. The composition of fungal cell walls also varies by taxonomy [94]. For instance, Zygomycetes feature a chitosan–glucan complex in their cell walls, while *Euascomycetes*, *Homobasidiomycetes*, and *Deuteromycetes* predominantly contain a chitin–glucan structure [94,95]. Based on industrial data, fungal byproducts represent an abundant yet underutilized resource for sustainable fungal chitin and chitosan production. Annually, over 80,000 tons of *Aspergillus niger* waste is generated as a byproduct of citric acid production [96]. In addition, 50,000 t of *Agaricus bisporus* waste is produced from mushroom farming [96,97].

Chitosan is present in high concentrations in several mushroom species, reducing the need for extensive deacetylation compared to its conversion from chitin in insects or crustaceans [96]. Furthermore, fungal biomass generally has negligible mineral content, meaning that pre-treatment steps to remove mineral residues are typically unnecessary [98].

While conventional methods for chitin extraction from crustaceans and fungi have been extensively reviewed [92,99], this review emphasizes insect-based approaches, particularly in the context of greener processing strategies.

To provide a basis for comparison, the following section outlines the conventional chitin extraction and chitosan production methods from insects, including common pre-treatment steps (e.g., defatting), core process stages (demineralization, deproteinization, deacetylation), and subsequent treatments such as bleaching.

The raw chitin-rich material is commonly washed, dried, and ground. Particle size can influence chitin extraction efficiency; thus, sieves are often employed to sort different sizes [100].

Certain insect species contain substantial amounts of fat. This high amount is a key distinction from crustaceans, which lack significant fat. The lipid components in insects may initiate hydrophobic reactions, leading to potential disruptions in the interactions between the chitin-rich parts of insects and chemical substances [49]. Therefore, a defatting protocol is often necessary. This procedure involves alcoholic and/or organic solvents such as n-hexane, acetone, chloroform, petroleum ether, and methanol [34]. However, defatting may not be necessary for raw materials from industry-side streams, such as oil extraction flakes [85] or cuticles. If defatting is not required, the primary conventional process of insect chitin extraction begins with acidic demineralization. This step involves the use of inorganic or organic acids (commonly used acids include hydrochloric acid (HCl), sulfuric acid, acetic acid, formic acid, and nitric acid) [101]. Abidin et al. (2020) state that the most used conditions involve HCl at 0.25–4 M [101]. Temperatures typically range from 21 to 100 °C for 15 min to 48 h, with solid-to-solvent ratios between 1:9 and 1:50 (*w*/*v*) [101].

**Table 2:** Comparative analysis of chitin and chitosan production from crustaceans, insects, and fungi.

Parameter	Crustaceans	Insects	Fungi
Availability	Seasonal, influenced by breeding cycles, molting periods, and fishing regulations.	Always available due to controlled farming systems.	Always available from year-round cultivation and agro-industrial byproducts.
Chitin/Chitosan Content in Raw Material (% Dry Weight)	Chitin: 6–75 [91,92].	Chitin: 1.2–60 [91].	Chitin + Chitosan: 2–42 [91,94].
Chitin Yield after Conventional (conv.) Processing (% of Dry Biomass)	Higher yields due to larger chitin content in raw material (15–40%) [102].	Generally lower yields from whole-body or larvae compared to crustaceans (5–15%); certain residues (e.g., sheddings, pupae) can yield comparable or higher amounts (up to ~55%) (see Table 1).	Generally lower than conventional sources [103].
Industry Scale	Industry standard.	Underexplored.	Underexplored.
Use of Waste Products	Only byproducts such as shrimp shells and crab processing waste are used.	Includes farming byproducts like BSF cocoons, silkworm pupae, mealworm sheddings, exoskeletons, and residues from protein/lipid extraction.	Agro-industrial residues (e.g., mushroom stalks, fruit bodies, mycelium, further biomass).
Defatting (conv.)	Not necessary due to low lipid content.	Often required for high-fat insects; not required for specific waste materials (e.g., sheddings).	Not required; fungal biomass has negligible fat content.
Demineralization (conv.) and Deproteinization (conv.)	Intensive demineralization due to high mineral content in raw materials.	Highly dependent on raw material. Intensive demineralization is needed for waste products like exuviae and cocoons (high mineral content).	Demineralization is not required due to negligible mineral content. Deproteinization is conducted under milder conditions due to reduced protein complexity [104].
Chitosan Production (via conv. Deacetylation)	Comparable to insects. Milder conditions for β-chitin; some α-chitin require harsher setups.	Comparable to crustaceans.	Typically, milder due to the inherent properties of fungal chitosan; chitosan is already present in some species.
Bleaching (conv.)	Well-established and less challenging due to more uniform and predictable pigment profiles.	More demanding due to diverse and complex pigments (e.g., melanins, catechols), requiring more intensive and tailored bleaching protocols.	Depending on raw material. For some, minimal bleaching is needed due to naturally low pigment levels [105].
Alternative Greener Methods	Well-studied with emerging green methods. Needs scaling.	Underexplored, high potential; greener methods need development and scaling.	Underexplored, high potential due to waste-based feedstocks and simpler extraction/modification.

An example of an explicit study on byproducts is provided by the work of Machado et al. (2024), which investigated chitin extraction from rearing residues of mealworm, superworm (*Zophobas morio*), and dubia cockroach (*Blaptica dubia*) [87]. As a first step, demineralization was performed for a 5 g dried sample using 100 mL of 1 M HCl, at 60 °C for 1.5 h [87]. During the acidic process, a slight discoloration of the biomass can occur due to the release of catechol compounds [69]. But, the chemical composition and structure of chitin can be negatively influenced by hydrochloric acid [69]. Prolonged or overly concentrated acid treatment can hydrolyze polymers and reduce their molecular weight [106]. Thus, Hahn et al. (2020) formulated a general guideline: the more intensive the demineralization process (in terms of pH, temperature, and duration), the greater the extent of hydrolysis [69,107].

The factors for the acidic process (time, concentration, temperature, and solution-to-solid ratio) applied in the extraction from insects are generally milder than in crustaceans [101]. Less stringent conditions are required due to the lower mineral content in insects (typically less than 10%) compared to crustacean shells (20–40%) [101]. It is important to note that certain waste materials of insects, such as cocoons and sheddings, may have significantly higher mineral content. Soetemans et al. (2020) demonstrated this, observing much higher ash content in BSF cocoons (19.4%) and BSF sheddings (24.5%) [40].

When considering the variable mineral content, an ongoing debate exists regarding whether the demineralization step is necessary for all insect materials. In the study conducted by Jantzen da Silva Lucas et al. (2021), which focused on insect cuticles, the demineralization step was skipped and deemed unnecessary [30]. This decision was based on the sample’s low mineral content. The cuticles exhibited an ash content of approximately 3.7%. Interestingly, a significant reduction in chitin yield was observed in another study using exoskeletons as a byproduct of insect protein production [108]. After the demineralization, a significant amount of the sample (40.7%) was lost. However, the reduction in ash content was insufficient, decreasing only from 3.6% to 2.8%. This slight improvement did not justify the quantity of acid used or the large volume of water required for demineralization [108].

Following demineralization, removing proteins through deproteinization is the next critical step. Various chemicals, such as NaOH, Na_2_CO_3_, NaHCO_3_, Na_2_SO_3_, Na_3_PO_4_, NaHSO_3_, Na_2_S, KOH, K_2_CO_3_, Ca(OH)_2_, and CaHSO_3_, serve as reagents for chemical deproteinization [34]. Reaction conditions vary significantly across studies; for detailed specifics, readers are encouraged to consult the relevant review articles [34,101,109]. For conventional practices, NaOH is typically employed at different concentrations, 0.125 to 5.0 M, at various temperatures (up to 160 °C), and durations (up to 30 h) [34,109]. Machado et al. (2024) provided an example for the extraction of rearing residues of mealworm, superworm, and dubia cockroach [87]. Following demineralization, deproteinization was carried out on an initial 5 g sample using 100 mL of 1 M NaOH at 100 °C for 20 h [87].

Deproteinization (DEP) before demineralization (DEM) can reduce chitin yield [83]. A possible explanation is that deproteinization erodes the protein layer covering the chitin matrix, leaving chitin more exposed to the acidic treatment, thus significantly increasing its hydrolysis [83].

Another study observed that chitin extracted using a demineralization–deproteinization treatment exhibited a higher yield [88]. Further distinctions could be analyzed, such as a higher crystallinity index and viscosity of DEM-DEP compared to chitin, which was obtained using DEP-DEM [88]. Intensifying alkaline treatment conditions, such as increasing incubation times, sodium hydroxide concentrations, and temperatures, correlate with enhanced deproteinization [108]. However, the alkaline treatment induces biopolymer hydrolysis and the partial deacetylation of chitin [34]. These unwanted reactions can reduce the molecular weight of the product [34].

It is difficult to select optimal parameters for protein purification because of the diverse life stages and various insect compositions.

In a study conducted by Soetemans et al. (2020), the determined chitin content in sheddings from the BSF was significantly higher when using a gravimetric method (31.3%) compared to a glucosamine-acetate-based quantification method (23.7) [40] (see Table 1). The chitin content determined gravimetrically indicated impurities. This issue became evident after a more extensive deproteinization process, which reduced the gravimetric chitin content to 26.6%, aligning more closely with the value obtained by the glucosamine-acetate-based method [40]. These findings suggest that purifying inevitable byproducts from insects for protein extraction can be more intricate than for other sources.

Raw chitin is commonly further processed with a bleaching step to remove pigments, particularly melanin complexes, and enhance purity [34]. Khatami et al. (2024) indicated that the bleaching step for insects is more challenging than for crustacean sources [65]. This difficulty is attributed to the presence of different structural colors and pigments involved in the process of cuticular tanning and sclerotization [65]. Decolorizing agents, such as hydrogen peroxide, potassium permanganate, and hypochlorite solutions, are frequently used [34]. Additionally, mixtures or pure solutions of methanol, ethanol, acetone, and/or chloroform are employed [34,109].

While chitin can be the final product, producing chitosan requires an additional deacetylation step. During deacetylation, chitin’s linked acetyl groups are removed and substituted with reactive amino groups [109]. Conventional chemical deacetylation is achieved with a strong NaOH solution. Specific parameters vary significantly across studies. Readers are therefore directed to comprehensive reviews [34,69,101,109]. An example of a technique for byproducts is provided by Machado et al. (2024), who investigated residues of mealworm, superworm, and dubia cockroach as potential chitosan sources [87]. Deacetylation of the extracted chitin was performed using 50 mL of 60% (*m*/*v*) NaOH, under conditions of 100 °C for 20 h [87].

However, differences in the deacetylation of α-chitin and β-chitin have been observed [110]. It was proposed that α-chitin can be transformed into chitosan using a 40–50% aqueous alkali solution at 100 to 160 °C for several hours [110]. In contrast, β-chitin deacetylation occurs at considerably lower temperatures compared to α-chitin. Around 80 °C is sufficient for achieving the deacetylation of β-chitin while avoiding an unwanted coloration process [110]. In other studies, it was reported that temperatures of at least 80 to 90 °C are required to provide the necessary activation energy for deacetylation, irrespective of the polymeric form of chitin [108,111].

Maintaining a low temperature and a reduced solvent/liquid ratio has increased chitosan yield and viscosity [108]. Lower temperatures and shorter reaction times during deacetylation lead to the production of chitosan with a reduced degree of deacetylation (DD) [108,111]. However, if the temperature fell below a specific threshold, chitosan yield decreased, while viscosity increased notably [108]. Nevertheless, prolonging the reaction time and applying harsher conditions during deacetylation can cause significant depolymerization and compromise specific polymer characteristics [30,112,113]. Thus, the optimal conditions for each setup must be determined before large-scale application.

Overall, there is a need to optimize the deacetylation process for converting insect chitin, as yields are comparatively lower than those from marine sources [108].

Following this, the conventional approach typically operates under severe conditions involving strong acidic (HCl) and alkaline (NaOH) solutions.

Currently, the handling of these chemicals post-extraction remains problematic. After neutralization, the combined effluents still contain high loads of dissolved solids and organic matter. Before release into the environment, they undergo conventional wastewater treatment on-site or municipal. Wastewater from demineralization, deproteinization, and deacetylation was first neutralized then subjected to sedimentation, biological treatment (activated sludge), and sand filtration prior to being discharged [114]. Inadequate waste management can lead to the release of these caustic substances into soil and water systems, causing severe ecological damage [114,115,116]. Furthermore, the process is energy-intensive, which often relies on fossil fuels [117]. This energy demand adds to the overall carbon footprint, especially in regions lacking access to renewable energy sources.

## 4. Green Chitin Extraction from Insects and Modifications to Chitosan

The utilization of corrosive and harmful chemicals is a significant drawback associated with the conventional methods for chitin extraction. These methods generate and discharge hazardous byproducts into our ecosystem [118]. Upcycling waste and byproducts into new resources must prioritize minimal environmental impact.

Green chemistry is a principle that decreases risks to humans and the environment by adapting synthesis, processing, and chemical use [119]. It was introduced mainly in the late 1990s and first defined by Anastas et al. (2009) as a “design of chemical products and processes to reduce or eliminate the use and generation of hazardous substances’’ [120]. Green chemistry can be considered a critical tool in the fight against climate change. A guiding framework is provided through the 12 principles of Green chemistry [121]. Defining the concept of greenness remains challenging. It encompasses various principles and standards, each with differing importance across academic and industrial fields.

In this review, the term “green methods” refers to environmentally conscious extraction techniques, while “sustainability” addresses broader system-level impacts such as energy use, waste generation, and economic viability.

Extracting chitin and converting it into chitosan involves several steps that can be adapted to greener methods. The complexity of these processes can pose significant challenges.

Mohan et al. (2022) extensively reviewed green methods for extracting chitin and chitosan from various resources [118]. Their focus did not include insect raw materials [118]. The investigated methods included “biological extraction” using microbial fermentation, “Enzyme-assisted extraction (EAE)”, “microwave-assisted extraction (MAE)”, “subcritical water extraction (SWE)”, “Ionic liquids (ILs) as well as (natural) deep eutectic solvents ((NA)DESs)”, and “ultrasonic-assisted extraction (UAE).”

A method is considered greener if it reduces or avoids the use of hazardous solvents such as HCl in both pre-and main processes. For example, chitin from BSF prepupae was extracted using a demineralization process that combined HCL with glycerol as a co-solvent [122]. Although information on chitin yield was not provided, making evaluation difficult, possible γ-chitin was identified [122]. A method employing citric acid as a demineralization agent at room temperature showed weak performance, achieving only 71% demineralization [123].

An advantage of using alternative greener acids or bases is their potential to lower environmental impact and reduce the degradation of chitin/chitosan [118]. However, a disadvantage is that these methods often demonstrate lower efficiency, as evidenced in the previous study and the studies presented in Table 3. Additionally, greater sustainability could be achieved by modifying pre-steps, particularly lipid and pigment removal. While green bleaching methods for insect biomass are still underexplored, advancements in chlorine-free agents like peracetic acid, tested via LCA in other fields, could be adapted for chitin bleaching [124].

**Figure 2:**
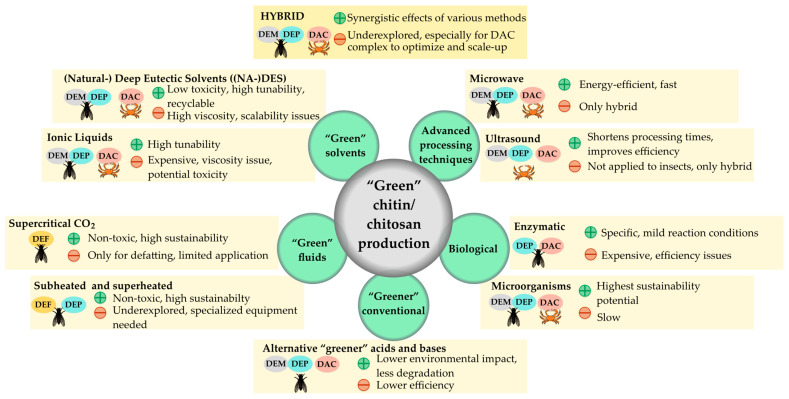
Green methods for chitin extraction and chitosan modification focusing on insect sources. Each method’s advantages (⊕) and limitations (⊖) are outlined. This figure illustrates the specific processes each technique is applied to demineralization (DEM), deproteinization (DEP), deacetylation (DAC), or defatting (DEF). Fly icons represent methods applied to insects, while crab icons indicate approaches primarily explored in crustaceans.

The following subchapters discuss greener extraction methods for insect chitin and their modifications in detail. Key findings are summarized in Table 3 and illustrated in Figure 2, highlighting their advantages and drawbacks.

### 4.1. Biological and Enzyme-Assisted Methods

Biological and enzyme-assisted methods offer greener alternatives to traditional chemical processes. Biological methods utilize natural agents, such as fermentation products like organic acids and proteases from microorganisms (primarily bacteria and fungi). These agents perform key steps, including demineralization, deproteinization, and deacetylation [96]. Biological methods are particularly interesting because they have major advantages: They use milder reaction conditions, reduce energy and chemical consumption, and avoid most toxic reagents [96]. Moreover, the easy recovery of enzymes, proteins, and pigments is an attractive attribute [125,126]. Based on these advantages, biological methods offer the significant benefit of having the highest sustainability potential among all extraction methods.

However, there are significant disadvantages that need to be overcome. Challenges include extended processing times for processes based on microorganisms, which is a limitation that warrants attention [127]. Additionally, issues around purity persist, as the substantial residues of proteins, pigments, and other impurities are often observed [30,128,129]. These challenges, as demonstrated in the following studies, highlight the need for further optimization to enhance efficiency, scalability, and product quality.

Alternatively, biological methods may directly employ enzymes, such as proteases for deproteinization and chitin deacetylases for deacetylation [130]. Enzymes provide additional benefits, including accelerated action time compared to fermentation and enhanced specificity [131].

Enzymatic deproteinization, combined with conventional chemical demineralization and deacetylation, yields chitosan with similar degrees of deacetylation obtained through chemical preparations [132]. However, the molecular weights are comparatively higher [132]. One study explored a green method alongside a conventional chemical process for deproteinization and demineralization [83]. Deproteinization was carried out through protease-producing bacteria (*Bacillus subtilis* and/or *Pseudomonas aeruginosa)*. Demineralization was achieved using lactic acid-producing bacteria (*Lactobacillus plantarum*). Co-cultivation of these three bacterial strains resulted in the highest chitin yield (11.85%), comparable to chemical extraction (10.18%) and significantly higher than the yields of separate fermentations (7.78–9.47%) [83]. However, conventional deacetylation of the obtained chitin led to a low chitosan yield, attributed by the authors to polymer depolymerization [83]. The authors did not propose a specific alternative to improve the yield. Nevertheless, potential strategies could include optimizing deacetylation conditions, such as reducing reaction temperatures and times or exploring enzymatic deacetylation methods to minimize polymer degradation [130,133].

To our knowledge, combined ultrasound methods have not yet been adapted for insects. However, another study integrated fermentation with low-intensity ultrasound extraction, focusing on crab shells [125]. In this context, low-intensity ultrasound (<1 W/cm^2^) enhanced fermentation efficiency for crustacean extraction, increasing decalcification by 16.72% and deproteinization by 33.45%. It also reduced the total fermentation time to 48 h by stimulating microbial growth. This stimulation enhanced enzyme activity and accelerated the breakdown of calcium carbonate and proteins [125]. These findings suggest a potential for adapting this method to the processing of insect biomass. Furthermore, the authors noted that protein hydrolysates (amino acids and peptides) and calcium lactate from fermentation residues could be utilized as feed supplements. This dual benefit could reduce costs and improve waste treatment efficiency [125]. The results underscore the potential of this approach for efficient and relatively rapid chitin extraction through *L. paracasei* fermentation.

Mealworm exoskeletons were used for chitin extraction through fermentation [134]. Screening of the mealworm surface led to the isolating of two strains identified as *Serratia marcescens* (1 VA) and *Serratia liquefaciens* (16 VB). They showed the highest protease activity of 96.78 and 97.34 U/mL, respectively. First, fermentation was accomplished with inoculated Tryptic Soy Broth (TSB) with single colonies of isolates *S. marcescens* (1 VA) and *S. liquefaciens* (16 VB) at 25 °C for 5 d. An alternative second fermentation was carried out with *Lactobacillus plantarum* (DSM 20 174) for 7 d, 30 °C. All processes achieved a demineralization rate of 94%. Nevertheless, mealworms generally contain relatively low mineral amounts. Chitin yields were similar for the 1 VA process at 28.2% and 16 VB at 28.6%. Chitin yields of the second fermentation method were comparatively lower. Challenges included a discolored final product containing catechol-compounded residues, sclerotin-like proteins, and pigments [134].

Jantzen da Silva Lucas et al. (2021) employed enzymatic hydrolysis for chitin extraction from mealworm cuticles [30]. An alcalase enzyme was deproteinized at 50 °C for 250 min [30]. Interestingly, the demineralization step was skipped due to the low mineral content of mealworm cuticles and the drastic yield reduction observed when applied. Chitin was obtained with a yield of 70.9% (by dry weight). Here, deacetylation to chitosan was carried out using a conventional alkaline. The overall yield of the process (cuticle-to-chitosan) was 31.9%. The obtained chitosan would require a higher degree of deacetylation for commercialization, as the degree was analyzed at 53.9% [30]. In this study, the applied enzyme was efficient (85% deproteinization efficiency). A protein residue of 8.3% was still observed [30]. After further processing into chitosan, the protein residue was reduced to 5.4%. The authors suggested that specific proteins might be sterically unavailable due to chitin protection [30]. Overall, the study produced promising results from a sustainable perspective since waste products like cuticles were used, and significantly fewer harmful chemicals and energy were applied for the extraction. However, purity still needs improvement.

Another example was presented in a study conducted by Wu (2011) focusing on chitosan preparation from *Clanis bilineata* (Striped Hawkmoth) larvae skin using endo- and exopeptidase for deproteinization [135]. Optimal hydrolysis conditions were found at pH 6.5 and 50 °C. Conventional demineralization and deacetylation achieved a chitosan yield of 37.37% with a high degree of deacetylation (93.2%). In contrast to the work of Jantzen da Silva Lucas et al. (2021) [26], this study found much lower protein residue in chitosan (0.81%). The discrepancy could stem from the use of different insect species. Another possible reason is the harsher conditions applied in the experimental setup.

Caligiani et al. (2018) focused on protein extraction rather than chitin [128]. An enzymatic approach was selected in addition to two different methods. Four enzymes were employed for hydrolyses, namely, *Bacillus licheniformis* protease, pepsin, papain, and pancreatin. The resulting pellet from each approach after hydrolysis contained chitin and a significant residue of unhydrolyzed proteins. Chitin yield was not calculated. Overall, despite using a rapid and greener method that avoids organic solvents and acidic/alkali solutions, the extraction efficiency for chitin was still insufficient [128].

To the authors’ knowledge, no green enzyme-assisted methods have been applied to deacetylate chitin to chitosan in insects. However, one study introduced a green approach for producing chitosan from chitin using bacterial chitin deacetylase from another resource [136]. For this purpose, a chitin deacetylase-producing strain was selected. The study built upon a previous green method, where chitin was extracted from *Litopenaeus vannamei* (Pacific White Shrimp) shell through sequential treatment with lactic acid (of *Bacillus coagulans* L2) and protease (*Alcaligens faecalis* S3). The extracted chitin was subsequently incubated at 30 °C with the newly isolated *Alcaligenes faecalis* CS4. This process resulted in chitosan with a 19.04% yield and 71% solubility [136]. The chitosan had a degree of deacetylation of 74.9%, a crystallinity index of 21.16%, and a molecular weight of 246.4 kDa [136]. Nevertheless, the experiment did not compare the performance of the studied agents against other enzymatic systems such as pepsin, papain, or trypsin.

### 4.2. Methods Combined with Microwave Techniques

Microwave-assisted techniques offer a greener alternative to conventional heating methods by accelerating chemical reactions with shorter processing times [118]. Unlike conventional heating, which relies on surface heat transfer mechanisms, microwave-assisted extraction enables direct internal heating by converting electromagnetic energy into thermal energy through molecular interactions with the electromagnetic field [137]. Microwave-based techniques also increase yields, enhance product purity, and improve properties through mechanisms like dipolar polarization and ionic conduction [118]. The rapid heating and reduced extraction time inherent to MAE contribute to its energy efficiency by minimizing the time and resources needed for processing [137].

When microwave approaches are combined with green solvents in hybrid processes, they hold great potential for improving sustainability while offering operational benefits [79,138]. Overall, the advantages of MAE methods include energy efficiency, significantly reduced processing times, and enhanced yield and product quality. However, as evidenced by the studies presented in this sub-chapter, these methods commonly rely on a hybrid process. Thus, harmful chemicals are often still involved, limiting their feasibility and sustainability as a standalone application.

Similarities in structures, morphologies, and chemical compositions between products obtained through conventional heating and microwave-assisted extraction have been described by Mohan et al. (2022) [118]. A study found that regenerated α-chitin, dissolved via microwave irradiation, exhibited a slightly lower molecular weight than untreated α-chitin [97]. This suggests that biopolymer depolymerization may occur during the process. While this could be beneficial when a low-molecular-weight polymer is desired, it poses challenges in other contexts. One study on molts of the spider Antilles Pinktoe Tarantula (*Caribena versicolor*) used a hybrid approach by combining chemical methods for deproteinization with MAE [79]. A short processing time of 3 min was achieved, though precise information on chitin yield was not provided. Although the process relied on conventional harsh chemicals (HCl and NaOH), the shorter reaction time should reduce energy consumption, offering a greener approach. As no data are available, further investigation is needed to confirm this potential benefit. Another example for crayfish combined a microwave-assisted method with deep eutectic solvent (DES) extraction [138]. This approach reduced solvent use and energy consumption [138].

### 4.3. Extraction with Supercritical, Superheated, and Subcritical Fluids

Supercritical, superheated, and subcritical fluid extractions are innovative techniques increasingly recognized for their contributions to green chemistry [86,139].

Supercritical fluids, such as carbon dioxide (SC-CO_2_), exist in a single phase when their critical temperature and pressure points are reached [140]. This state creates a solvent with unique properties, combining a viscosity akin to gases with a density comparable to liquids [141]. The SC-CO_2_ technique was applied in the chitin extraction process in insects [142,143]. Until now, it was used for fat removal as a pre-step rather than as a part of the main process. An investigation by Sipponen et al. (2017) [143] demonstrated that SC-CO_2_ defatting effectively removed 79% of lipids from *Acheta domesticus* (house cricket) and 74% from *Tenebrio molitor* (mealworm). The extraction residues contained only 4.8% and 3.5% lipids, respectively [143]. In the work conducted by Laroche et al. (2019), fat removal was performed via SC-CO_2_ for two insects (mealworm and house cricket) [142]. SC-CO_2_ achieved a lipid yield of 22.1 ± 0.6% for mealworms, comparable to Soxhlet ethanol extraction (28.8 ± 5.9%) and hexane extraction (25.5 ± 0.1%). For house crickets, SC-CO_2_ extracted significantly less fat (11.9% ± 1.4%) compared to ethanol (22.7 ± 2.9%) but was comparable to hexane (14.6 ± 0.1%). While SC-CO_2_ shows promise as a green method, its application should be further explored, particularly as ethanol, another viable and green solvent, also demonstrates high efficiency for fat extraction [144].

Superheated fluids are liquids heated above their boiling points under pressure [145]. This state enhances their ability to dissolve and extract target compounds, and the need for harsh chemicals is minimized [145]. Superheated water hydrolysis of BSF exuviae for chitin extraction was used, and the resulting chitosan was applied to the textile sector [86]. This work applied a green superheated water method to deproteinization [86]. However, this study was combined with Soxhlet extraction for fat removal with diethyl ether and included conventional demineralization and deacetylation. A yield of 20% chitin was achieved, and protein was extracted in a water medium at neutral pH. The extracted protein could potentially be utilized after sterilization [86].

Subcritical fluids, mainly water, exist at conditions below their critical point but above their boiling point [146].

Subcritical water demonstrates promising extraction efficiency for certain biomolecules, such as lipids and proteins, and is often regarded as a green alternative [146]. A study used subcritical water extraction for BSF farming wastes for lipid and protein removal to produce biodiesel production [147]. The aqueous phase contained hydrolyzed proteins with a low molecular mass around 6 kDa [147].

Although superheated, supercritical, and subcritical fluids are not yet widely used for comprehensive chitin extraction or its modification to chitosan, their application in insect-derived chitin processes is steadily growing, as highlighted in this review. The presented superheated, supercritical, and subcritical processes offer significant advantages due to their non-toxic nature and improved sustainability [141]. Drawbacks result from their limited application and the need for specialized equipment [141]. Nonetheless, they represent an underexplored but promising area for green and efficient insect biomass valorization.

### 4.4. Ionic Liquids (ILs), Deep Eutectic Solvents (DES), Natural Deep Eutectic Solvents (NADESs), and the Role of Artificial Intelligence (AI)

Ionic liquids (ILs) are a prominent topic in green chemistry, frequently used in the extraction and modification of chitin, with most applications focusing on crustaceans. Composed of cations and anions with ionic bonding, ILs can exhibit a liquid state at or near room temperature [148]. Beyond ionic bonding, ILs exhibit several interactions, such as van der Waals forces or hydrogen bonding [148].

Despite their growing attention and potential, ionic liquids (ILs) face several significant drawbacks that limit their broader application. Some ILs may pose environmental risks and have application limitations due to potential toxicity issues [129,149].

In response, research has increasingly shifted toward deep eutectic solvents (DESs), a related class of solvents that share similar properties but often present improved sustainability. Of particular interest are natural deep eutectic solvents (NADESs), a subclass of DESs composed of naturally derived, biodegradable components, making them especially promising for green extraction processes.

Sulthan et al. (2023) compared ILs and DESs in chitin extraction processes [150]. DESs consist of a eutectic system of Lewis or Brønsted acids and bases that can incorporate diverse anionic and/or cationic components [151]. They exhibit low melting points upon mixing, a phenomenon occurring below the individual melting point of each component [83]. Natural DESs (NADESs) are composed of naturally occurring compounds, such as organic acids, sugars, and amino acids [152]. In the context of sustainability, emphasis should be directed towards NADESs.

While it is asserted that the interaction of DESs on chitin remains unknown [150], Sharma et al. (2013) proposed a possible mechanism [153]. The authors suggested that the dissolution of Chitin in DESs predominantly stems from the disruption of existing hydrogen bonding and the formation of new hydrogen bonds [153]. Drawing from the work of Sharma et al., 2013 [153], it was proposed that the acidic HBD may react with minerals to release cations, water, and carbon dioxide (demineralization) [154]. This interaction is believed to result in a less dense protein–chitin fibril network [154]. Simultaneously, HBAs were presumed to establish new hydrogen bonds with protein and chitin, disrupting chitin–protein fibrils (deproteinization) [154]. According to this hypothesis, chitin dissolution in DESs occurs by blocking intra- and inter-molecular hydrogen bonds in chitin [154]. This process leads to new hydrogen bonds between chitin and DESs [154]. Thus, a limited quantity of chitin is soluble in DESs [153]. To increase the yield, the dissolved chitin must be recovered by adding ineffective solvents such as ethanol or water [154].

Understanding the DES mechanisms remains an area of ongoing research. Further insights into DES interactions with chitin from different insect species and matrices are necessary to achieve a fundamental understanding of the mechanism. Consequently, a critical evaluation of ionic liquids in terms of sustainability is required [155]. Key challenges include decomposition and volatility, hygroscopicity, toxicity, and biodegradability [148]. Other concerns involve flammability, impurities, energy consumption costs, synthesis, regeneration, and low renewability [148]. Currently, significant preparation costs further hinder their widespread application [67]. Despite these challenges, the field is advancing, with solutions actively explored. Chen et al. (2021) proposed 13 strategies to address these limitations and improve the sustainability of ILs [148]. An advantage of IL is the tunable properties, as their specific characteristics depend on the choice of anion and cation composition [156]. Similarly, for NADESs, combining hydrogen bond acceptors (HBAs) and hydrogen bond donors (HBDs) in varying molar ratios offers great turnability. DESs and NADESs have clear advantages in contrast to ILs. They are considered environmentally sustainable, non-toxic, economically viable, tunable, and easily synthesized without purification [150]. Another big plus is its high recyclability [150]. The solvent recycling of DESs offers dual advantages, both economic and environmental.

However, a significant drawback of NADESs is that, although their sources are relatively low cost, the large quantities required for an optimized mass ratio pose a significant cost challenge [154]. Their high viscosity, an issue also related to ILs, causes further complications [157,158]. Therefore, more systematic research is needed to unveil the correlation between the mass ratio of the chitin source and the DES and its impact on yield and purity [154].

Different approaches for chitin and chitosan processes have been developed to produce polymers with tailored molecular weights. For example, Wineinger et al. (2020) developed an approach using ILs to enhance chitin yield while regulating molecular weight for the molts of *Litopenaeus vannamei* (Jumbo shrimp) [159]. Dong et al. (2023) demonstrated the use of IL mixtures to extract chitin from the shells of *Penaeus vannamei* (white shrimp) [160]. In this study, five different IL mixtures were synthesized and applied, namely, 1-ethyl-3-methylimidazole acetate ([EMIM][Ac]) and 1-butyl-3-methylimidazole bromide ([BMIM][Br]) in ratios of 0:1, 1:1, 3:2, 4:1, and 1:0 [160]. Demineralization was performed with organic acid (citric acid). Interestingly, the yield of chitin extracted using ILs was approximately twice as high (35.72 ± 0.31%) compared to the acid–base method (17.50 ± 0.16%). Additionally, the whiteness index (WI) of chitin obtained with the IL (EMIM][Ac] [BMIM][Br] 3:2) (94.93 ± 0.09) exceeded that of commercially available chitin (93.31 ± 0.11). Moreover, surface morphology, structure, and thermal stability resembled commercially available chitin [160]. Both studies presented encouraging findings with potential applications for insect species.

Building on the potential of DESs and NADESs as greener alternatives, recent studies have begun exploring their application in insect chitin processing. One NADES study design for chitin preparation was developed for insects (BSF) to investigate the selectivity of deproteinization and demineralization [129]. The study investigated how the pH and pKa values of the NADES correlate with demineralization, deproteinization, and the crystallinity indexes of the extracted chitin. The authors found that acidic NADESs with a basic HBA and an acidic HBD, as well as alkaline NADESs with an acid HBA and alkali HBD, were more effective for producing chitin. Choline chloride (ChCl)-organic acid NADESs exhibited superior performance in decalcification [129]. It was attributed to their acidity, which facilitated protein dissolution, and their unique bonding network, including hydrogen bonds [129,161,162,163]. The analysis did not show any link between the NADESs’ pH and yield, purity, or the degree of deacetylation of chitin [129]. Furthermore, no correlation was evident between the acidity of the HBD and chitin’s purity [129].

NADESs were also reused: Even after being recycled thrice, the demineralization and deproteinization capacities of NADESs did not show a notable decline [129].

A practical study design that combined various green methods for a comprehensive analysis of house crickets was presented by Psarianos et al. (2022) [123]. In addition to conventional chemical control methods, different enzymes were applied for deproteinization, incorporating microwave, organic acid, and lactic acid fermentation. Moreover, a one-pot approach involving a DES and fermentation was implemented [123]. Citric acid demineralization exhibited the lowest performance, with lactic acid fermentation showing promise [123]. The combination of conventional and microwave methods produced the most favorable results [123].

Chitin content post-deproteinization led to suboptimal performance for all greener methods. The conventional method with NaOH surpassed greener ones by more than double [123]. Insect-specific properties like robust protein–chitin networks may have led to limitations [123]. The biological method produced chitosan with the lowest molecular weight (86.8 × 10^3^ g/mol) due to enzymatic depolymerization by bromelain [123]. The antioxidant activity of chitosan produced by the biological method (49.3 ± 5.2%) was slightly lower than that from the chemical method (59.0 ± 0.6%). The difference was not statistically significant [123]. The enzymatic method is a promising alternative for producing chitosan with comparable high antioxidant properties. However, although it contributes to improved sustainability, its limited extraction efficiency remains a limitation.

The biological method involving bromelain and lactic acid has been successfully employed for chitin extraction at scale (2 L) [123]. Despite this success, the subsequent conversion to chitosan relied on traditional chemical deacetylation using 50% NaOH at high temperatures (130 °C) [123]. A critical gap remains in developing fully green methods for chitin-to-chitosan conversion.

Another work conducted by Li et al. (2023) on crayfish showed a hybrid method for chitin production employing a microwave-assisted extraction with deep eutectic solvents [138]. This study reported favorable results. Varying DES types and mass ratios influenced the final purity and yield of the extracted chitin. The optimized conditions involved the DES choline chloride/lactic acid (with a molar ratio of 1:10) and a DES-to-crayfish sample mass ratio of 10/1. Processing under these conditions for 30 min at 120 °C produced chitin with a yield of 19.11% and a purity of 97.44% [138]. Further information can be found in Table 3. Recycling occurred with the successful reuse of DES four times. The authors emphasize a significant challenge in simultaneously achieving higher yield and greater purity [138].

A recent study demonstrated the potential of ternary deep eutectic solvents as a green extraction method, highlighting their low viscosity, recyclability, and efficiency at ambient temperature [164]. Conducted on *Litopenaeus vannamei* (shrimp) shell powder, the result showed a designed DES made from three components (N-methylacetamide, N-methylurea, and acetic acid in a molar ratio 1:1:3) that exhibited a comparatively low viscosity (7.38 mPa·s at 25 °C) and melting point of 16.82 °C [164]. The extraction efficiency at room temperature was described as “remarkably high”, including a deproteinization efficiency of 92.67% and a demineralization efficiency of 99.07% with a shell-to-DES ratio of 1:30 and 48 h extraction time. The yield of the extracted chitin was not further specified. The novel DES demonstrated robust recyclability with consistent viscosity over repeated cycles. After 10 cycles, demineralization and deproteinization rates stayed at 89.78% and 86.84%, respectively [164]. To the author’s knowledge, green strategies have not been used to deacetylate chitin to chitosan in insects up to this point. Evidence suggests that deep eutectic solvents simultaneously induce surface modification, such as deacetylation to chitosan [165]. One approach presented a green method for obtaining chitosan from commercially available chitin with a combination of NADESs [166]. In this study, the amount of NaOH could be reduced to a concentration of 25 wt % NaOH. Chitosan was prepared with 83.77% deacetylation degree when combined with the NADES of betaine and glycerol (molar ratio 1:2.5) for 12 h at 100 °C [166]. Interestingly, using NADESs provided two key advantages: it improved the degree of deacetylation and minimized the molecular-weight reduction typically caused by alkali depolymerization.

While DES and NADES methods have demonstrated promising sustainability profiles, optimizing their formulation for specific applications remains a challenge. Integrating advanced technologies, such as AI, into these processes can streamline this optimization, enabling more targeted extraction parameters, higher yields as well as purity, and reduced resource consumption. AI is the science and engineering of creating intelligent machines and computer programs that can perform tasks that typically require human intelligence [167].

AI systems are particularly interesting in IL and NADES study designs because their high tunability can be adjusted and improved by AI systems. For example, AI can facilitate the development of customized NADES formulations tailored for the specific chitin extraction requirements of diverse insect species and matrices. A review that delves into modeling the physicochemical attributes of DESs shows how such an adaptation could be realized [168]. Beyond solvent optimization, AI can address broader challenges in green chitin and chitosan production. Machine learning algorithms can analyze vast datasets to predict optimal extraction conditions, including temperature, time, and solvent composition [169]. This predictive capability significantly reduces trial-and-error experimentation, streamlining process optimization.

AI also plays a crucial role in enzyme discovery and optimization strategies. For example, the Preoptem deep learning tool was applied to identify a thermophilic chitinase from marine metagenomic datasets, demonstrating its utility in accelerating enzyme discovery for chitin degradation [170]. However, a similar AI-driven process or study design could be adapted to discover or optimize deacetylases from organisms like fungi, which naturally produce these enzymes. Then, such deacetylases could be applied to insect-derived chitin to efficiently produce chitosan.

### 4.5. Emerging Green Processes and Hybrid Methods

Building on the advancements in ILs and NADESs, emerging green methods offer additional avenues for innovation. While many greener methods have been studied, various greener methods, such as electrochemical or ultrasonic methods, remain unexplored in insect applications. Also, lastly, the combination of different greener methods can lead to synergistic effects and should be further explored. These combinations are often referred to as hybrid methods. While possible synergistic effects present a significant advantage to be explored, they remain underexplored [125,171]. In addition, they pose challenges for industrial applications due to their increased complexity and the need for specialized setups [171,172]. As presented in Table 3, some studies are already applying this approach.

**Table 3:** Green chitin extraction techniques employed on insects with a few extraordinary examples on different resources. Conventional control methods are not included.

Insect and Material	Process	Deproteinization	Demineralization	Deacetylation	Results	Green Aspects	Ref.
*Acheta* *Domesticus* (House Cricket)	Enzymatic, microwave, greener acids, fermentation, DES	1. Papain 24 h, 60 °C. 2. Bromelain 5 h, 60 °C.	1. MW: 1 M HCl; 500 W, 8 min. 2. Citric Acid 0.5 M, room temp, 2 h. 3. Lactic acid fermentation; 30 °C, 48 h.	Conventional	Demineralization performance: Lowest performance: citric acid. Ferm. with lactic acid promising. Microwave and control (conventional): Best performance, conventional better than lactic acid, worse than MW. Chitin content after deproteinization: “greener” methods’ lowest performance. Conventional (NaOH) more than double. Biological process led to lowest molar mass (depolymerization). Effective large-scale production (2 L) of chitosan with biological method (bromelain + lactic acid)	DF 0 DEM + DEP + DA - B 0 REC 0	[123]
		One pot: 1. Fermentation with *B. subtilis* 5 d, 37 °C. 2. DES (Choline Chloride/Malonic Acid), 80 °C, 3 h.					
BSF pupal shell waste	Fermentation	Protease-producing bacteria (*Bacillus subtilis* and *Pseudomonas aeruginosa*) 37 °C, 5 d.	Lactic acid-producing bacteria (*Lactobacillus plantarum*), 37 °C, 5 d.	Conventional	Chemical and biological extraction yielded chitin at 10.18% and 11.85%, respectively. Maximum chitosan yield of 6.58% based on chitin mass.	DF 0 DEM + DEP + DA - B 0 REC 0	[83]
Crab shell	Fermentation + ultrasound-treatment	*Lacticaseibacillus paracasei* 37 °C, 48 h with low-intensity ultra sound treatment (0.167 W/cm^2^) for 10 min at 8 h intervals.		n.s.	Improved decalcification (DEC) by 16.72% (DEM rate: 71.77%) and deproteinization by 33.45% (DEP rate: 59.50%). Fermentation time shortened to 48 h. Chitin’s molecular structure preserved; deacetylation degree unchanged when combined with low-intensity ultrasound.	DF 0 DEM + DEP + DA 0 B 0 REC 0	[125]
*Tenebrio molitor* (Meal-worm) cuticle	Enzymatic	Alcalase enzyme; pH 8.0, 50 °C, 250 min.	Skipped, since low mineral amount.	Conventional	Enzyme used was efficient (85% DEP efficiency but protein residue: 8.3%) Sufficient reduction justifies use of green process. Rest mineral content: 3.7%. Cuticle-to-chitosan yield of 31.9%. Higher degree of deacetylation needed as DD of chitosan is 53.9%.	DF 0 DEM 0 DEP + DA - B 0 REC 0	[30]
*Clanis bilineata* (Lined Hawk- moth) larvae skin	Enzymatic	Endo- and exo-peptidases (flavourzyme); 40–60 °C, 8 h.	Conventional.	Conventional	Optimum parameters for outcome at pH 6.5 and 50 °C. Chitosan yield 31.37% based on raw mass. Protein residue in chitosan 0.81%. Only green deproteinization. Rest conventional.	DF 0 DEM - DEP + DA - B 0 REC 0	[135]
BSF prepupae	Enzymatic	(1.) *B.·licheniformis* protease pH 6.5, 60 °C, 16 h. (2.) Pepsin pH 3.0, 37 °C, 16 h. (3.) Papain 60 °C, pH 7.5, 16 h. (4.) Pancreatin 37 °C, pH 7.8, overnight.	n.s.	n.s.	Hydrolytic activity: highest for *B. licheniformis*, followed by pancreatin, followed by papain. Pepsin lowest performance. Degree of hydrolysis (DH %): ~6% (*B. licheniformis*), 17% pepsin, up to ~25% for pancreatin and papain. Chitin yield (~9%) with residues of minerals and non-hydrolyzed proteins.	DF 0 DEM 0 DEP + DA 0 B 0 REC 0	[128]
*Caribena* *versicolor* (Tarantula) molts (ecdysis cuticles)	Microwave-assisted	Hybrid: Chemical 2.5 M NaOH+ MW * 750 W 2450 MhZ reached 95 °C, 3 min.	Skipped	n.s.	No fully green bleaching and defatting step, but combination with microwave treatment. Thus, faster and expected to be greener. Chitin content at 19% of the molt by molt dry mass.	DF + DEP + DEM 0 DA 0 B + REC 0	[79]
Crayfish shell waste not insect	Hybrid: MW + DES	1. Choline chloride/lactic acid (CL). 2. Choline chloride/urea (CU). 3. Choline chloride/glycerol (CG); 80–140 °C 10–40 min with microwave radiation = 300 W with different weight ratios of sample to DES.		n.s.	Extraction yield (YE), chitin yield (YC), and purity: CL: 19.11%/73.22%/97.44%. CG: 53.40%/88.17%/41.99%. CU 49.16%/88.87%/45.97%. Ash content: CL: ca. 0%, CG: 56.48%, CU: 52.41%. Protein content: CL: 2.56%, CG: 1.53%, CU: 1.62%. Recycling of DES.	DF 0 DEP + DEM + DA + B - REC +	[138]
BSF farming waste	Subcritical water extraction	n.s.	n.s.	n.s.	Defatting: Subcritical water extraction of lipids. Optimized conditions (236.8 °C, 10 min, 1 g/100 mL) resulted in a lipid yield of 13.31% by total sample weight. Aqueous phase contains proteins (hydrolyzed, with low molecular masses of 6 kDa).	DF + DEM 0 DEP + DA 0 B 0 REC 0	[147]
BSF exuviae	Superheated water	Superheated water; 150 °C, 1.5, 10, 15, 20 h.	Conventional.	Conventional	Soxhlet extraction with diethyl ether 7% fat, 40% proteins, and 20% chitin.	DF - DEP: + DEM - DA - B 0 REC 0	[86]
*Penaeus* *vannamei* (White shrimp) shell not insect	Binary ionic liquids	Different ionic liquids based on [EMIM][Ac] * with [BMIM][Br] * mixed at ratios of 0:1, 1:1, 3:2, 4:1, and 1:0; 110 °C, 24 h.	Citric acid 12% (*w*/*v*); 60 °C, 5 h.	n.s.	Optimal chitin quality obtained with IL (3:2) at viscosity, conductivity, and radius of gyration values of 0.16 ± 0.00 Pa s, 0.30 ± 0.01 S/m, and 0.10 ± 0.00 nm, respectively. Chitin yield using IL reached up to 35.72 ± 0.31%, about twice as high as acid–base method (17.50 ± 0.16%). Whiteness index (WI) of chitin extracted by IL (3:2) (94.93 ± 0.09) superior to commercial chitin (93.31 ± 0.11). Surface morphology, secondary structure, and thermal stability comparable to commercially available chitin.	DF 0 DEP + DEM + DA 0 B: + REC 0	[160]
BSF prepupae	Co-solvent	Conventional.	Co-solvent of glycerol and HCl (37% glycerol, 5% HCl); 90 °C, 2 h.	n.s.	Defatting: via Soxhlet and petroleum ether. No info on chitin yield. γ-chitin identified.	DF - DEM + DEP - DA 0 B - REC 0	[122]
BSF exo- skeleton	Enzymatic, “greener” acids, fermentation, SC-CO_2_, special defatting.	(1.) + (2.) Protease from *Bacillus licheniformis;* 37 °C, 72 h.	(1.) + (2.) EtOH (for DF), lactic acid, and acetic acid; 25°C, 27h.	n.s.	Defatting via EtOH, optional *Pseudomonas fluorescens* (lipase production), or SC-CO_2_. Four methods applied: (1.) DEM+DF then DEP (Chitin: 69.4 ± 1.0%). (2.) DEP first, then DEM+DF (Chitin: 65.4 ± 0.2%, best protein, and lipid removal efficiency: protein decrease 33%, lipid reduction 38.5%). (3.) SC-CO_2_ + fermentation (Chitin: 69.8 ± 1.2%). (4.) Acetic acid + EtOH then fermentation (Chitin: 72.4 ± 1.6%, highest yield). High chitin yields suggest possible residual impurities as noted by authors.	DF + DEM + DEP + DA 0 B 0 REC 0	[173]
		(3.) SC-CO_2_ (defatting (65 °C, 2 h, 5400 psi)) then *Lactobacillus plantarum*, *Bacillus subtilis*, and *Pseudomonas fluorescens* (30 °C, 5 d).					
		(4.) *L. plantarum*, *B. subtilis*, and *P. fluorescens* (30 °C, 5 d).	(4.) Acetic acid (50 mL), 70% ethanol (100 mL) (1 day).				
BSF prepupae, skimmed	NADES	Different NADESs: HBD (ChCl and betaine) and HBA (DL-lactic acid, n-butyric acid, glycerol, urea and oxalic acid); 50–80 °C, 2 h.		n.s.	Complex results on purity, yield, and DD. Lacking link between pH of NADESs and yield, purity, or DD of chitin. No correlation between acidity of HBD and chitin’s purity. Relatively high yield for several NADES applications in comparison to conventional method. Degree of deacetylation of chitin from samples relatively high in comparison to commercial sample. Recycling of NADES.	DF 0 DEM + DEP + REC + B 0 DA 0 REC +	[129]
Mealworm exo-skeletons	Fermentation with isolates from mealworm	1st fermentation: Inoculated TSB with colony-forming units of isolates from mealworm with *Serratia marcescens* (1 VA) and *Serratia liquefaciens* (16 VB); 25 °C, 5 d. Alternative 2nd fermentation: *Lactobacillus plantarum* (DSM 20 174); 30 °C, 7 d.		n.s.	Two varieties, *S. marcescens* (1 VA) and *S. liquefaciens* (16 VB), demonstrated best protease activity, reaching 96.78 U/mL and 97.34 U/mL, respectively. Demineralization of around 94% for all processes. Chitin Yields (dry weight): 1 VA: 28.2%; 16 VB: 28.6%; 1 VA+ *L. plantarum*: 18.6%; 16 VB + *L. plantarum:* 17.2%. Discolored product with residues of catechol compounds, sclerotin-like proteins, and pigments.	DF 0 DEM + DEP + DA 0 B 0 REC 0	[134]
*Litopenaeus* *Vannamei* (Shrimp) shell powder not insect	DES (+MW)	1. N-methyl urea, N-methylacetamide, and acetic acid (1:1:3), different mass ratios of shrimp shell powder to DES (1:10, 1:20; 1:30), MW, 3–11 min. 2. N-methylurea, N-methylacetamide, and acetic acid (1:1:3), different mass ratios of shrimp shell powder to DES (1.10, 1:20; 1:30), RT, 6–48 h.		n.s.	Innovative DES with notably lower melting point (−16.82 °C) and viscosity (7.38 mPa·s; 25 °C) alongside high extraction efficiency at room temperature. Demineralization rate up to 99.07%. Deproteinization rate up to 92.67%. DD: 7.89% for conventional method; 6.77% for DES method at room temperature. 6.39% for DES method combined with MW. DD for conventional method’s chitin higher since NaOH used with intense deacetylation effects at elevated temperatures demonstrated robust recyclability, with DES showing consistent viscosity over repeated cycles. After 10 cycles, demineralization and deproteinization rates stayed at 89.78% and 86.84%, respectively.	DF 0 DEM + DEP + DA 0 B 0 REC +	[164]

* 1-ethyl-3-methylimidazole acetate ([EMIM][Ac]) and 1-butyl-3-methylimidazole bromide salt ([BMIM][Br]); n.s.—not stated; MW—microwave; B—bleaching; DF—defatting; DEP—deproteinization; DEM—demineralization; DA—deacetylation; REC—recycling; BSF—black soldier fly; -—indicates an unsustainable character; 0—indicates not assessable due to lack of data or the process step not applied; +—indicates a green method; SC-CO_2_—supercritical CO_2_ extraction; IL—ionic liquid; DES—deep eutectic solvent; NADES—natural deep eutectic solvent; CL—choline chloride/lactic acid; CU—choline chloride/urea; CG—choline chloride/glycerol; WI—whiteness index; HBD—hydrogen bond donor; HBA—hydrogen bond acceptor; DD—degree of deacetylation; RT—room temperature; TSB—tryptic soy broth; EtOH—ethanol; and Ref.—reference.

## 5. Limitations

**Figure 3:**
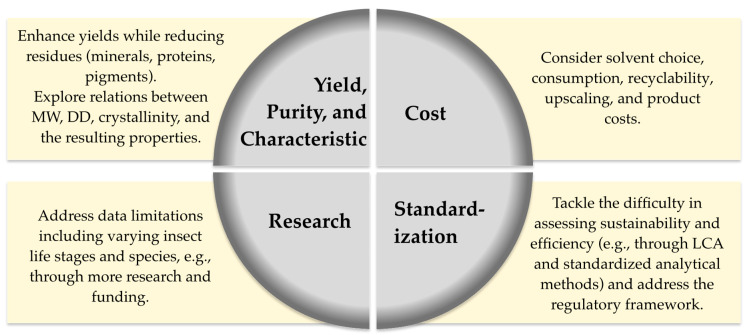
Key challenges and focus areas for green insect chitin and chitosan production (MW—molecular weight; DD—degree of deacetylation; and LCA—life cycle assessment).

Despite the great potential of chitin extraction and its modification to chitosan from insects, several challenges remain to be overcome. Several limitations hinder its extraction, modification to chitosan, and subsequent utilization in a sustainable manner (Figure 3).

Insects’ unique characteristics lead to challenges in chitin extraction, mainly when novel green methods with limited research and data are employed. A high fat percentage in some insect species leads to additional defatting steps using extra resources. Moreover, the high content of pigments present in insects also exacerbates this issue. It is important to note that, through the co-occurrence of multiple life stages during the rearing process, the selection of a specific life stage for processing is hindered [63]. The impact of feed on chitin yield was observed in the black soldier fly larvae [84]. This underscores the need for further research into how insect feed affects chitin content and quality.

Chitin yields may be overestimated due to residues included in yield determinations when using gravimetric methods [49]. The difficulty in removing proteins and achieving pure extracted chitin underscores the importance of refining green extraction and modification processes.

The methods discussed in this review, while promising, come with inherent challenges that limit their broader application.

Chemical methods often involve the use of harsh acids and alkalis, which can degrade chitin properties and generate hazardous wastes [62]. Biological methods face issues with the quality of chitin when residues of protein, for example, are too high [30,128,129].

Greener methods face common challenges when scaling up from laboratory setups to industrial applications.

The economic viability and the pivotal transition from laboratory-scale operations to large-scale industrial production demand careful consideration and optimization.

Biological methods, for example, are often limited by slow processing times, making them less competitive at an industrial scale [127]. Similarly, NADES and IL approaches require high quantities of solvents and face issues with viscosity and recovery that complicate large-scale implementation [154,157,158]. Hybrid methods, such as combinations of greener processes with microwave or ultrasound, present opportunities for synergistic effects but require specialized setups and pose challenges in terms of complexity [125,171,172]. Techniques like superheated, supercritical, and subcritical solvents also demand significant resource investment and tailored infrastructure [141,174].

Another factor is chitosan’s relatively high cost on the market. It makes the biopolymer less competitive compared to materials with similar attributes. Unique biological activities exclusive to chitosan (e.g., antimicrobial properties) could be identified to increase its value and justify its costs [101].

However, once these challenges are overcome, costs can be diluted into industrial-scale production, making production cost-effective and suitable for replacing traditional methods that are more harmful to the environment.

Assessing the sustainability of chitin extraction and modification processes remains difficult due to the lack of standardized metrics. Notably, life cycle assessments (LCAs) have not been widely implemented for chitin/chitosan processes in insects. Only a few research studies have been published that evaluate the impact of insects on food and feed production systems [175], but none of them are related to chitin/chitosan production. An LCA approach for insect protein was demonstrated by Halloran et al., 2016 [16]. This absence makes it difficult to evaluate the actual sustainability of these processes.

Therefore, benchmarks from crustacean-derived processes can serve as a reference. Environmental assessments and LCAs of chitosan production from crustacean sources have revealed significant environmental impacts, primarily due to high chemical usage and energy consumption [176,177,178]. For example, a study on chitosan production in India and Europe reported that producing 1 kg of chitosan required approximately 1.4 kg of chitin, 5.18 kg of NaOH, 250 L of water, 31 MJ of wood fuel, and 1.06 kWh of electricity [114].

A comparative environmental impact assessment of three chitin extraction and chitosan production pathways from shrimp shells showed that environmental impacts varied widely depending on the method, with greenhouse gas emissions ranging from 184 to 1891 g CO_2_-eq per kilogram of chitosan [177]. Eco-solvent-based extraction exhibited the lowest overall environmental burden, and additional sustainability gains were achieved through the valorization of waste shrimp shells.

A life cycle assessment of a marine shell-based biorefinery producing chitosan from lobster shells found that sodium hydroxide contributed to 42% of the total human health-related environmental burden, while substituting it with potassium hydroxide reduced that impact to 17% [176].

Thus, there is an urgent need to apply green metrics such as LCAs to insect-derived chitin/chitosan processes to obtain an honest assessment of the environmental impact.

Even after implementing greener methods, the overall sustainability must be thoroughly evaluated, particularly regarding greenhouse gas emissions. The carbon footprint associated with all chitin extraction methods, whether green or not, should be carefully evaluated. This is particularly relevant for the demineralization and deacetylation processes, where CO_2_ is generated during the reaction. Notably, the demineralization of 1 kg of chitin from crab shells through acidic processes yields 0.7 to 0.9 kg/kg of CO_2_, equivalent to 70% of its initial weight [179]. Similar data for insects are lacking, highlighting a knowledge gap that needs to be addressed.

The circular economy could provide concepts for utilizing CO_2_ in other processes. For instance, CO_2_ generated during chitin extraction could be employed and captured in algae production or other CO_2_-intense applications [180].

Without such empirical data, sustainability claims remain unsubstantiated and prone to “greenwashing”, a growing concern in the rapidly expanding bioeconomy. Regulatory frameworks, such as the EU Taxonomy for Sustainable Activities and Product Environmental Footprint (PEF) methodology, are emerging tools aimed at standardizing environmental claims [181,182,183]. However, implementing these frameworks on a global scale remains a significant challenge. Given the complexity, cost, and expertise required for conducting full assessments, regulatory pressure will likely be essential to ensure industry compliance and to avoid the unchecked use of sustainability labels. Future research should focus on developing streamlined LCA tools, harmonized metrics, and practical policy support to facilitate responsible scaling and industrial application of insect-derived chitin/chitosan.

Lastly, regulatory challenges must be addressed to successfully commercialize insect-derived chitin and chitosan. In regions like the EU, insects are classified as Novel Foods, requiring extensive safety evaluations and product-specific authorizations before market approval [184]. Access to insect resources such as waste products is also more challenging [166]. However, certain insect species have already been approved, and further regulatory facilitation is expected. Globally significant amounts of insect waste exist. A significant opportunity for sustainable development is provided.

## 6. Conclusions

It is important to value chitin and its derivatives, such as chitosan, as a scarce and valuable resource that should be managed carefully with insects as a source. The field of green chitin extraction from insects remains in its early stages, and further research is necessary. Insect matrices pose unique challenges, demanding additional research across various species and life stages. Here, a particular focus should be directed to waste materials from insect production that are especially chitin-rich. With estimated high production amounts of insects for the near future, generated chitin-rich byproducts will be of special interest.

The variability in measuring chitin content and characterizing its properties poses significant challenges. Addressing these challenges is essential to ensure the production of high-quality chitin and chitosan products that are competitive and have minimal impurities. Reliable methods are essential to standardize these measurements and ensure consistency across studies. Additionally, there is a critical need for the development of green pre-processing techniques. They include defatting, bleaching, demineralization, and deproteinization, as well as greener deacetylation methods that are tailored explicitly for insect-derived matrices to facilitate the green production of chitin and chitosan.

Varying factors, such as the differing compositions of insect species, their life stages, and the types of waste materials, make it challenging to evaluate the potential of utilizing chitin and chitosan derived from insects. Further limitations arise when comparing insect material to conventional sources like crustaceans. Overall, the assessment of the sustainability of the technique is complex. Notably, the lack of real data and quantitative assessments based on LCAs or further green chemical metrics hampers a comprehensive understanding of the actual sustainability of these green methods. Nevertheless, it represents a promising field to be investigated to address the many knowledge gaps in the literature.

Future research should focus on developing more efficient green solvents, optimizing the extraction process to reduce energy consumption, and exploring high-value uses for byproducts generated during processing.

## Data Availability

No new data were created or analyzed in this study. Data sharing is not applicable to this article.
